# *Myo*-inositol mediates reactive oxygen species-induced programmed cell death via salicylic acid-dependent and ethylene-dependent pathways in apple

**DOI:** 10.1038/s41438-020-00357-2

**Published:** 2020-09-01

**Authors:** Lingyu Hu, Kun Zhou, Guijin Ren, Shulin Yang, Yuan Liu, Zhijun Zhang, Yangtiansu Li, Xiaoqing Gong, Fengwang Ma

**Affiliations:** State Key Laboratory of Crop Stress Biology for Arid Areas/Shaanxi Key Laboratory of Apple, College of Horticulture, Northwest A&F University, 712100 Yangling, Shaanxi China

**Keywords:** Plant development, Secondary metabolism

## Abstract

As a versatile compound, *myo*-inositol plays vital roles in plant biochemistry and physiology. We previously showed that exogenous application of *myo*-inositol had a positive role in salinity tolerance in *Malus hupehensis* Rehd. In this study, we used *MdMIPS* (the rate-limiting gene of *myo*-inositol biosynthesis) transgenic apple lines to gain new insights into the physiological role of *myo*-inositol in apple. Decreasing *myo*-inositol biosynthesis in apple lines by RNA silencing of *MdMIPS1/2* led to extensive programmed cell death, which manifested as necrosis of both the leaves and roots and, ultimately, plant death. Necrosis was directly caused by the excessive accumulation of reactive oxygen species, which may be closely associated with the cell wall polysaccharide-mediated increase in salicylic acid and a compromised antioxidant system, and this process was enhanced by an increase in ethylene production. In addition, a high accumulation of sorbitol promoted necrosis. This synergetic interplay between salicylic acid and ethylene was further supported by the fact that increased *myo*-inositol accumulation significantly delayed leaf senescence in *MdMIPS1*-overexpressing apple lines. Taken together, our results indicated that apple *myo*-inositol regulates reactive oxygen species-induced programmed cell death through salicylic acid-dependent and ethylene-dependent pathways.

## Introduction

*Myo*-inositol (MI) is a widespread and versatile metabolite in plants, animals, yeast, and microorganisms^1^. In higher plants, MI is incorporated into phosphatidylinositol phosphate, MI polyphosphates, and certain sphingolipid-signaling molecules that function in various processes, such as solute synthesis (galactinol and raffinose-family oligosaccharides)^2^, stress tolerance^3,4^, and regulation of cell death^5,6^. Moreover, the oxidation product of MI, d-glucuronic acid (GlcA), is further utilized for the synthesis of cell wall pectin and noncellulose compounds^7^.

MI biosynthesis involves three successive steps: (1) phosphorylation of glucose to glucose-6-phosphate (G6P) by hexokinase (HXK); (2) conversion of G6P to *myo*-inositol-1-phosphate by *myo*-inositol-1-phosphate synthase (MIPS); and (3) dephosphorylation of *myo*-inositol-1-phosphate to MI by *myo*-inositol monophosphatase (MIPase) (Fig. S1)^8^. MIPS is the rate-limiting enzyme in the biosynthetic pathway of MI and its derivatives, and its evolutionary profile suggests that it is an ancient protein^9–11^. Although yeast and animals contain only one *MIPS* gene^12^, plants possess multiple *MIPS* paralogs, suggesting a functional divergence of MIPS in plants^13^. For instance, Donahue et al. reported that three *MIPS* genes were expressed differently in *Arabidopsis thaliana* and suggested a key role of AtMIPS1 in MI biosynthesis^14^. The *atmips1* mutant, which is characterized by a reduced MI level, displays pleiotropic developmental defects, including reduced root growth and altered venation in its cotyledons. Moreover, a striking feature of the *atmips1* loss-of-function mutant is the light intensity-dependent formation of leaf lesions due to programmed cell death (PCD)^15,16^. A reduced MI level was also reported to result in pleiotropic phenotypes, such as advanced leaf senescence (a slow form of PCD), in MIPS-suppressed transgenic potato (*Solanum tuberosum* L.) plants^17^.

PCD is essential for plant growth and development and also plays a role in the response of plants to stress, such as pathogen infections^18,19^. Reactive oxygen species (ROS) such as hydrogen peroxide (H_2_O_2_) and superoxide ion (O_2_^−^) as well as the phytohormones salicylic acid (SA) and ethylene (ET) appear to be key factors in PCD regulation^15,20^. In plants, ROS are constantly generated in multiple cellular compartments, such as chloroplasts, mitochondria, and peroxisomes. Although the generation of ROS is rapidly triggered by various kinds of biotic stresses, ROS can also act as signaling molecules. Moreover, they are also toxic byproducts of aerobic metabolism. Excessive production of ROS leads to irreversible oxidative stress and, ultimately, cell death^21^. As a result, plants have evolved various ROS-scavenging mechanisms, including the production of enzymatic antioxidants, such as superoxide dismutase (SOD), catalase (CAT), ascorbate peroxidase (APX), and glutathione peroxidase (GPX) and the production of nonenzymatic antioxidants such as ascorbic acid (AsA) and glutathione (GSH)^21,22^. SA is a crucial plant hormone that mediates pathogen defense responses and leaf senescence and often interacts with ROS to regulate plant oxidative stress and cell death^23,24^. It has also been reported that SA can promote H_2_O_2_ accumulation by compromising the activity of antioxidant enzymes^23,24^. Furthermore, disruption in SA biosynthesis can prevent lesion formation in the *atmips1* mutant via an SA induction-deficient 2 (*sid2*) mutation^15^.

The gaseous phytohormone ET has vital roles in many aspects of plant growth and development^25,26^. As critical enzymes catalyzing the rate-limiting step in the ET biosynthetic pathway, both 1-aminocyclopropane-1-carboxylic acid (ACC) synthase (ACS) and ACC oxidase (ACO) are regulated at different levels by a complex signaling network^27–30^. In addition, ET production is associated with wounding, pathogen attack, anaerobiosis, senescence, and oxidative stress. ET has also been suggested to be a positive regulator of ROS production and a disseminator of cell death signaling (or senescence)^20,31^. However, it is unclear whether ET is involved in MI-mediated PCD in plants.

Apple (*Malus domestica*) is one of the most popular and culturally important fruit crop species worldwide. The significance of MI remains unknown in apple, although various roles of MI have been described in many other plant species. Previously, we reported that exogenous MI can promote plant growth and can alleviate salinity-induced stress in *M. hupehensis* Rehd^32^. In this study, we further investigated the physiological role of MI by altering the expression level of *MdMIPS1/2* in apple using a transgenic approach. Our data indicate that MI can directly promote the integrity of cell wall polysaccharides and can mediate ROS-induced PCD via SA-dependent and ET-dependent pathways in apple.

## Results

### MI biosynthesis is vital to apple viability

To identify *MIPS* genes in apple, the sequences of three *Arabidopsis* MIPSs, AtMIPS1 (AT4G39800.1), AtMIPS2 (AT2G22240), and AtMIPS3 (AT5G10170), were used as the queries for searching the Apple Genome Database v1.0^33^. Three candidates, MDP0000698835, MDP0000207103, and MDP0000459576, were identified and subsequently designated MdMIPS1, MdMIPS2, and MdMIPS3, respectively. The similarity was the highest between MdMIPS1 and the three AtMIPSs, and the highest similarity of the MdMIPSs was shared with AtMIPS3 (Fig. S2a, b). Alignment of the coding region sequences revealed that MdMIPS3 has a partial sequence corresponding to that of MdMIPS2 (Fig. S2a), suggesting that MdMIPS3 arose from a fragment duplication of MdMIPS2 in parallel with the events of apple whole-genome duplication^33^. *MdMIPS1* and *MdMIPS2* encode proteins of 509 amino acid residues; these genes share 95.37% and 96.47% identity in their coding region and amino acid sequence, respectively. Both MdMIPSs and AtMIPSs have a highly conserved pentapeptide in their core catalytic domain (Fig. S2a), suggesting that they have biochemically similar MIPS properties^34^. In addition, the results of reverse transcription-quantitative PCR (RT-qPCR) showed that the expression profiles of *MdMIPS1* and *MdMIPS2* in Royal Gala were similar, with both showing relatively high expression in the leaves and fruits (Fig. S2c). Thus, MdMIPS1 and MdMIPS2 functions are likely redundant in apple.

**Fig. 1:**
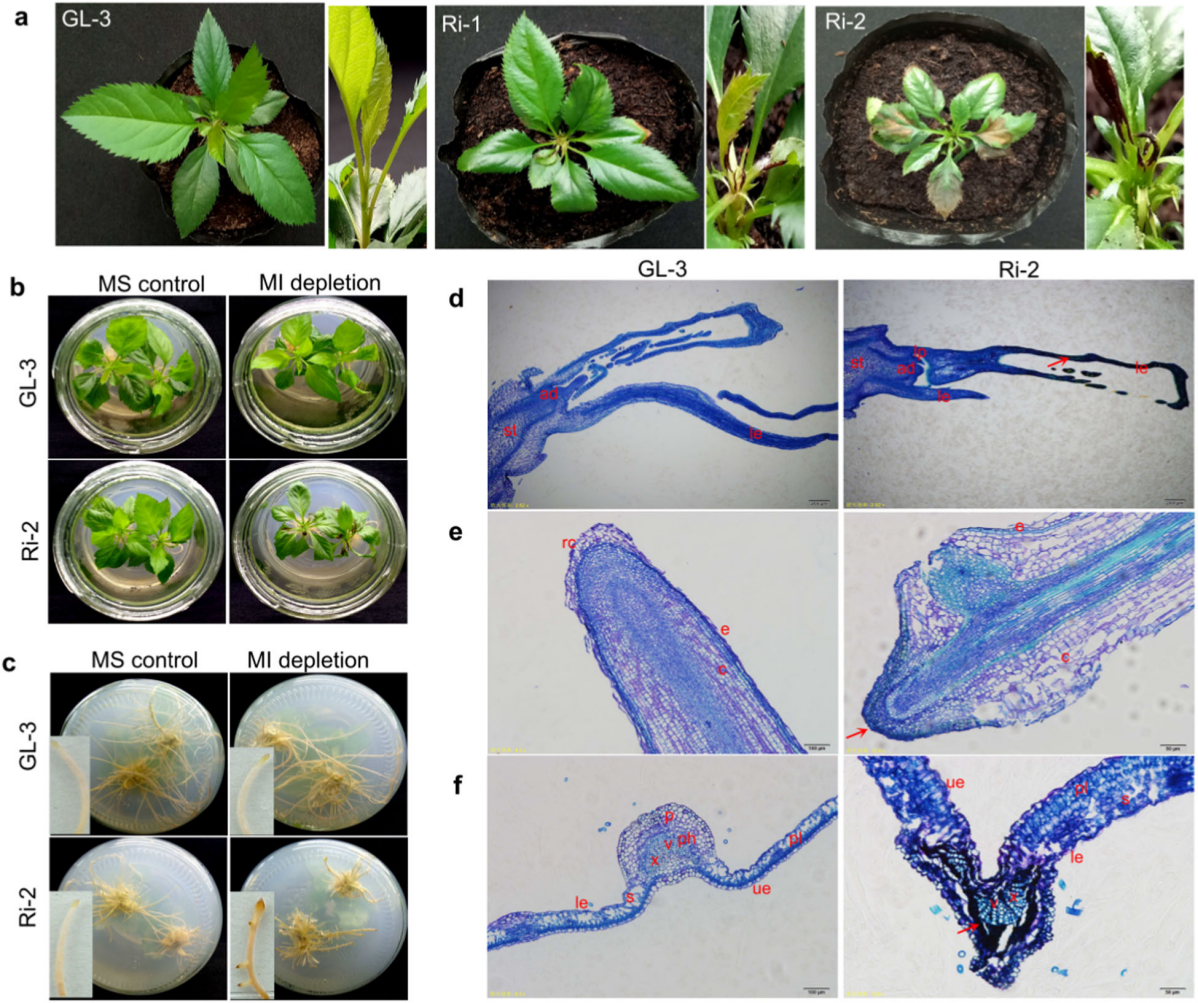
Phenotypes of MdMIPS1-RNAi apple lines with reduced *myo*-inositol biosynthesis. **a** Phenotypes of Gala-3 (GL-3) and MdMIPS1-RNAi apple lines under greenhouse conditions. **b**, **c** Assay results of MI depletion. Toluidine blue O staining of longitudinal sections of a shoot tip **d** and root **e** and cross-sections of a leaf **f** from GL-3 and Ri-2 under MI-depleted MS medium. MS control and MI depletion represent normal and MI-depleted MS media, respectively; le leaf, ad apical dome, lp leaf primordium, st stem, rc root cap, e epidermis, c cortex, le lower epidermis, p parenchyma, ph phloem, pl palisade cell, s spongy mesophyll, ue upper epidermis, v vessel, and x xylem. Bars = 200 μm **d**, 100 μm (**e** and **f** for GL-3), and 50 μm (**e** and **f** for Ri-2). The arrow indicates necrosis.

To elucidate the physiological role of MI in apple, we chose a 394-bp fragment from *MdMIPS1* to specifically silence both *MdMIPS1* and *MdMIPS2* by RNA interference (RNAi). Two silenced lines, Ri-1 and Ri-2, were obtained and cultivated normally in Murashige and Skoog (MS) media (Fig. S3). RT-qPCR analysis verified that both the *MdMIPS1* and *MdMIPS2* transcripts were significantly reduced in both RNAi lines (Fig. S3a, b). However, necrosis was visible on the leaves of Ri-1 and Ri-2 at 20 and 7 days after the seedlings were removed from tissue culture and transplanted into soil, respectively (Fig. 1a). Both RNAi lines eventually died.

The results of gas chromatography–mass spectrometry (GC–MS) analysis revealed a significant reduction in MI accumulation in Ri-1 (15 days old) under greenhouse conditions (Table 1).

**Table 1:** Soluble sugar profiles of GL-3 and transgenic apple lines (mg g^−1^ fresh weight).

	*Myo*-inositol	Sorbitol	Sucrose	Glucose	Galactose
(A)					
GL-3	0.156 ± 0.0112	5.275 ± 0.101	0.789 ± 0.028	0.620 ± 0.087	0.026 ± 0.005
Ri-1	0.024 ± 0.002***	9.394 ± 0.662***	1.034 ± 0.120*	0.681 ± 0.029	0.029 ± 0.005
(B)					
GL-3	0.100 ± 0.003	8.486 ± 0.228	1.127 ± 0.038	1.872 ± 0.317	0.030 ± 0.001
OE-4	0.200 ± 0.002***	7.902 ± 0.084*	0.603 ± 0.007***	1.807 ± 0.289	0.032 ± 0.005
OE-6	0.270 ± 0.003***	7.698 ± 0.127**	0.848 ± 0.019***	1.735 ± 0.341	0.026 ± 0.004

(A) 15 days old aerial parts and (B) 2 months old leaves. The data are the means ± SDs (*n* = 3, three biological replicates).

***P < 0.001; **P < 0.01; *P < 0.05.

The levels of both sorbitol and sucrose increased, which was indicative of decreased MIPS activity in the RNAi lines (Table 1). Necrosis rapidly occurred in both the leaves and roots of the RNAi lines, especially in Ri-2, under MI-depleted MS conditions (Figs. 1b, c and S3c). By contrast, *MdMIPS1*-overexpressing (OE) apple lines (OE-4 and OE-6) were characterized by increased MI accumulation and decreased levels of both sorbitol and sucrose, and both transgenic apple lines grew normally under MI-depleted conditions (Table 1 and Fig. S3). No significant differences were found for glucose in any of the transgenic apple lines (Table 1).

The results of toluidine blue O staining revealed that necrosis initially occurred in the young leaves and root caps but not in the meristems or primordium from Ri-2 (Fig. 1d, e), suggesting that the decreased MI level did not affect organ development but greatly disrupted subsequent growth of the RNAi lines. In addition, necrosis was largely found in the vicinity of the vein, which extended into the leaves of the RNAi lines (Fig. 1a, b and Fig. S3c). Cross-sections of leaves of Ri-2 revealed degradation of parenchymal cells in the parenchyma and phloem but not in the xylem vessels in the veins (Fig. 1f). Thus, MI biosynthesis is vital for normal apple growth.

### Necrosis is closely associated with ROS-mediated PCD in RNAi apple lines

**Fig. 2:**
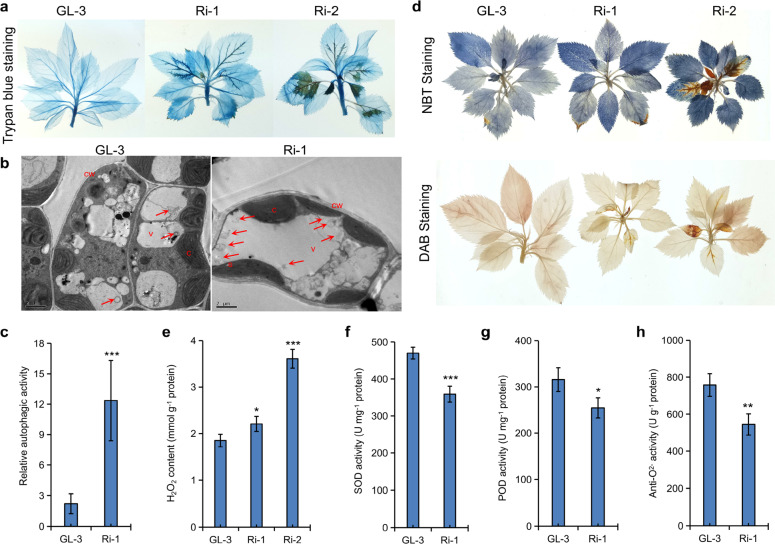
Analysis of PCD occurrence and ROS levels in MdMIPS1-RNAi apple lines. Trypan blue **a**, NBT, and DAB staining **d** and H_2_O_2_ levels in the leaves **e** of GL-3 and MdMIPS1-RNAi apple lines under MI-depleted MS conditions. Representative TEM images of cell structure **b**, relative autophagic activity **c**, and superoxide dismutase (SOD) activity **f**, peroxidase (POD) activity **g** and anti-O_2_^−^ activity **h** in aerial parts of GL-3 and Ri-1 under greenhouse conditions. The data are the means ± SDs (*n* ≥ 14 for **c**, more than 14 cells were used to quantify the structures; *n* = 3 for **e**, three biological replicates; *n* = 4 for **f**–**h**, four biological replicates). In comparison with GL-3, ***P < 0.001, **P < 0.01, and *P < 0.05. cw cell wall; c chloroplast; v vacuole; and s starch. The arrows indicate autophagic bodies. Bar = 2 μm for **b.**

Under MI-depleted MS conditions, trypan blue staining revealed severe cell death of the leaves of RNAi apple plants (Fig. 2a).

Transmission electron microscopy (TEM) showed vacuolar autophagy in normal leaves of Ri-1 (Fig. 2b). Vacuolar autophagy has been reported in plants undergoing senescence^35^. We also noted that there were up to four times as many autophagic bodies in Ri-1 than in GL-3, suggesting extensive autophagy (Fig. 2b, c). Furthermore, RNA sequencing (RNA-seq) analysis showed that the Gene Ontology (GO) enrichment of PCD involved in cell development (GO:0010623) was significantly enriched in Ri-1 (Table S1). Thus, it seems that necrosis was closely associated with severe PCD in the RNAi lines.

Both H_2_O_2_ and O_2_^−^ significantly increased in the RNAi leaves under MI-depleted MS conditions (Fig. 2d, e). RNA-seq analysis revealed two CuZn-SOD encoding genes, MD03G1128900 and MD11G1148200, that were significantly downregulated in Ri-1 (Table S2). However, no significant differences were observed for other ROS-scavenging antioxidant genes, such as *APX*, *CAT*, *GPX*, and ROS-producing enzyme gene respiratory burst oxidase homolog (*RBOH*), in Ri-1. Metabolic analysis showed that the levels of oxidized and reduced GSH and AsA were not significantly altered in Ri-1 (Table S3). However, the SOD activity significantly decreased in Ri-1, and the anti-O_2_^−^ activity and peroxidase (POD) activity were also reduced in Ri-1 (Fig. 2f–h), which is consistent with the role of ROS in cell death. Thus, our results indicated that necrosis was the end result of severe PCD, and it was closely associated with the high accumulation of ROS in RNAi lines.

### Abnormal cell wall polysaccharides may contribute to high SA accumulation in RNAi apple lines

**Fig. 3:**
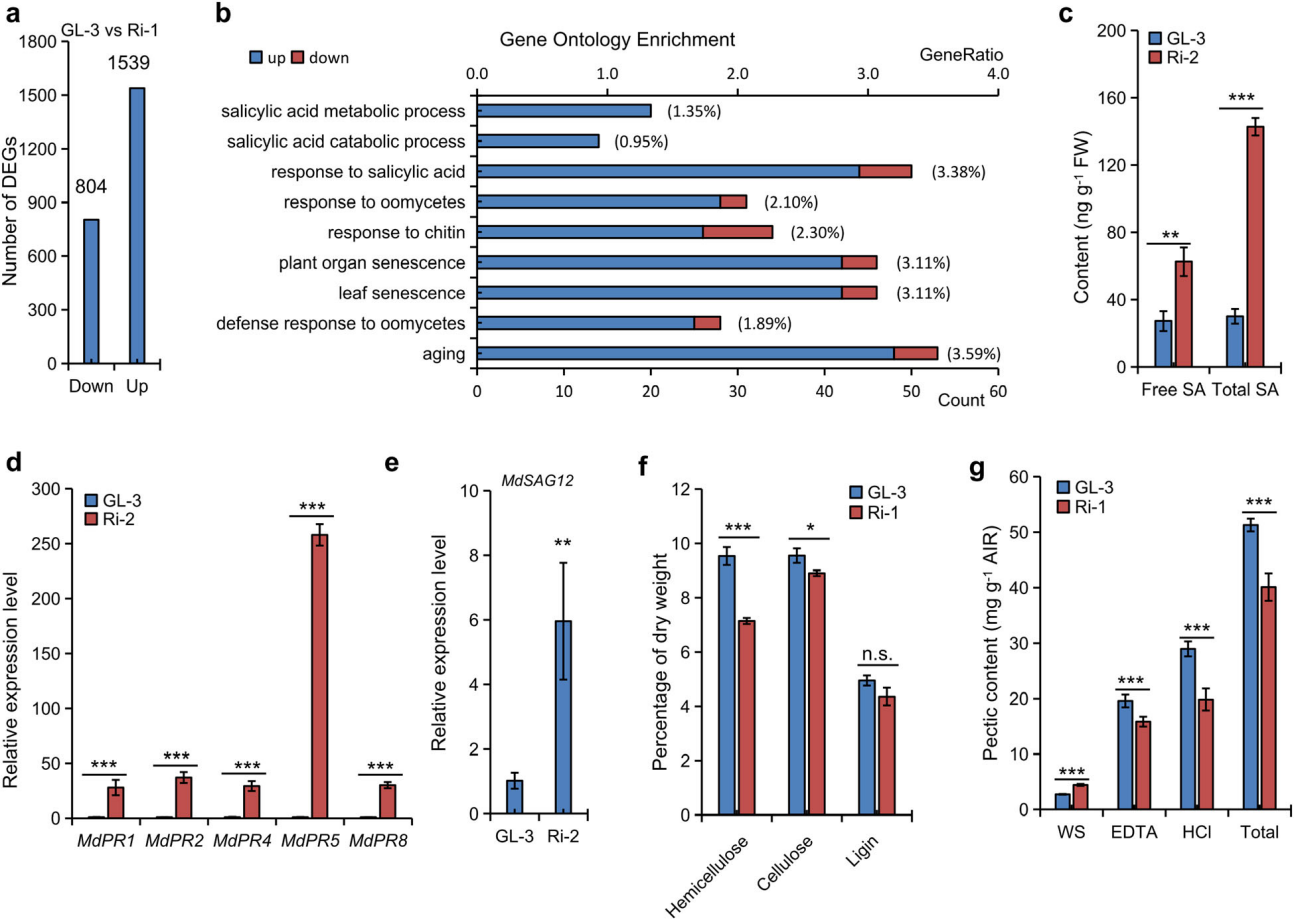
Analysis of SA accumulation in MdMIPS1-RNAi apple lines. **a** Number of upregulated and downregulated genes in GL-3 vs. Ri-1 by RNA-seq analysis. **b** Gene ontology terms were statistically enriched in DEGs and were associated with SA and senescence in Ri-1 (*P*_adj_ < 0.05). Levels of SA **c** and the expression levels of *MdPR*s **d** and *MdSAG12* **e** in the l**e**aves of Ri-2 and GL-3 under MI-depleted MS conditions. Composition of the cell wall **f** and pectin **g** in leaves of Ri-1 and GL-3 under greenhouse conditions (15 days old). The data are the means ± SDs (*n* = 3 for **c**–**f**, three biological replicates; *n* = 6 for **g**, six biological replicates). In comparison to GL-3, ***P < 0.001, **P < 0.01, and *P < 0.05. FW fresh weight. WS EDTA, and HCl represent crude cold water-soluble, EDTA-soluble, and HCl-soluble fractions, respectively.

The results of RNA-seq analysis revealed that 804 and 1539 genes were downregulated and upregulated in Ri-1, respectively (Fig. 3a).

These differentially expressed genes (DEGs) were significantly concentrated in SA-related biological processes, such as SA metabolism, senescence, and pathogen resistance, in Ri-1 according to GO-enrichment analysis (Fig. 3b and Table S1). In addition, SA-inducible *pathogenesis-related* (*PR*) genes, the SA biosynthesis regulatory gene *enhanced disease susceptibility 1* (*EDS1*), *phytoalexin deficient 4* (*PAD4*), and the senescence marker gene *senescence-associated gene 12* (*SAG12*) were significantly induced in Ri-1 (Table S2). Phytohormones and metabolic analyses revealed that SA and its derivatives, such as benzoic acid, 4-hydroxybenzoic acid, and phenyl salicylate, were significantly increased in Ri-1 (Table S3). *MdPR*s and *MdSAG12*, as well as the SA level, were significantly increased in Ri-2 leaves before necrosis occurred under MI-depleted MS conditions (Fig. 3c–e), suggesting that SA signaling was enhanced in the RNAi lines.

MI is critical for the synthesis of cell wall polysaccharides via the intermediate GlcA^7^, and cell wall polysaccharides have been demonstrated to be critical for SA-mediated host–pathogen interactions^36–38^. Thus, we analyzed the cell wall composition of the aerial parts from Ri-1 before the appearance of necrosis under greenhouse conditions. The levels of cellulose, hemicellulose, and total pectin were significantly reduced in Ri-1, but the lignin level was unchanged compared with that of GL-3 (Fig. 3f, g). TEM revealed that these changes did not induce cell wall breakdown in the leaves of Ri-1 (Fig. 2b). Moreover, further analysis revealed an approximate increase of 62.5% for cold water-soluble (WS) pectin in Ri-1. However, both EDTA-soluble and HCl-soluble fractions were significantly decreased (Fig. 3g). Increased WS pectin levels were previously reported to stimulate SA accumulation^37,38^. By contrast, no significant differences were found in either the pectic composition or the *MdPR* transcripts in the OE apple lines (Figs. S4 and S5). The levels of *MdPR* transcripts also increased in both the GL-3 and transgenic apple leaves under MI-depleted MS conditions (Fig. S5), indicating that MI deficiency can stimulate SA signaling in apple. Thus, it was concluded that high SA accumulation may be directly associated with abnormal cell walls in RNAi apple lines.

### ET bursts promote necrosis in RNAi apple lines

**Fig. 4:**
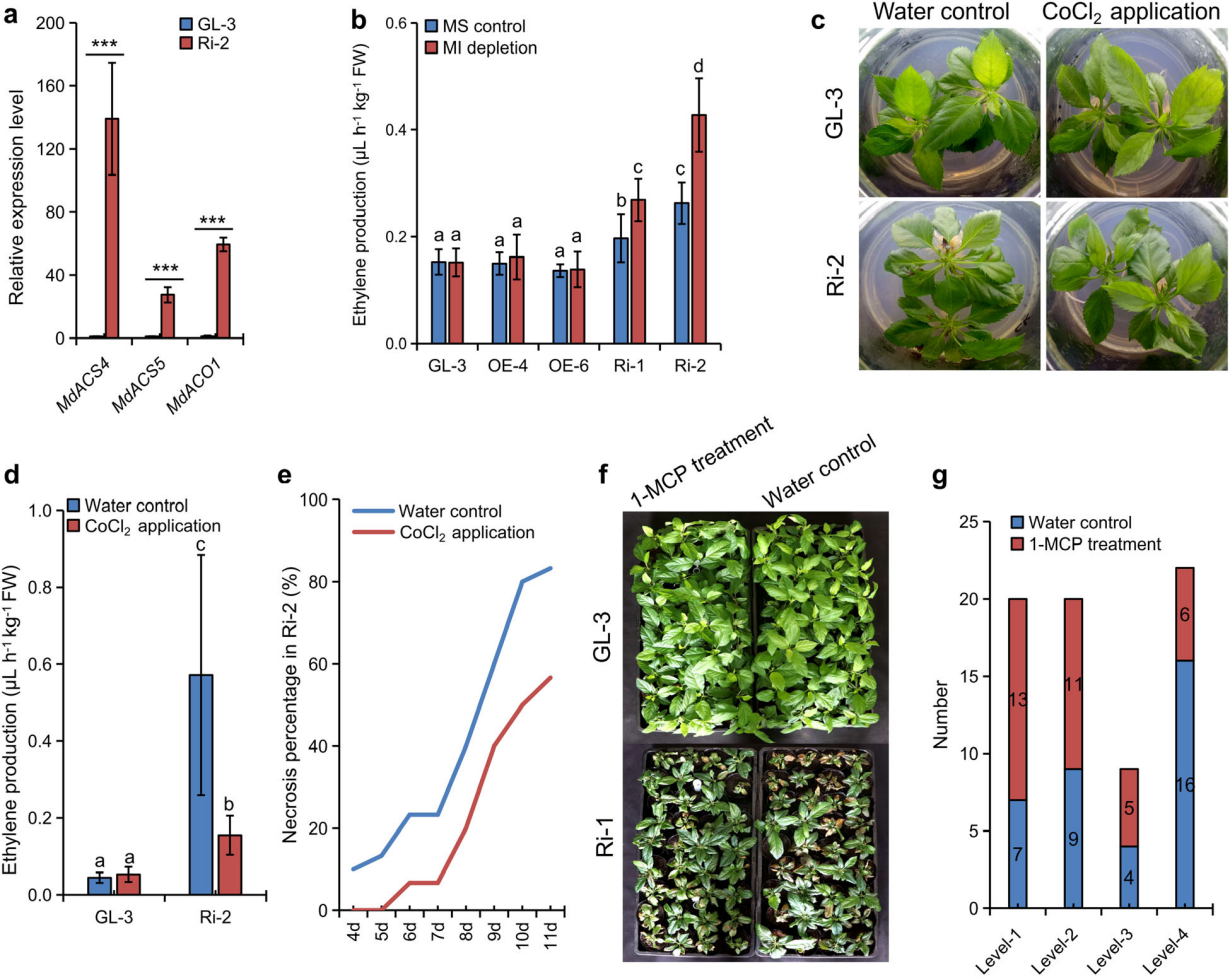
Analysis of ET production in MdMIPS1-RNAi apple lines. **a** Transcript levels of ET biosynthetic genes in GL-3 and Ri-2 leaves under MI-depleted MS conditions. **b** ET production in GL-3 and transgenic apple lines under tissue culture conditions. **c**–**e** Effects of CoCl_2_ application on GL-3 and Ri-2 under MS tissue culture conditions. **f** and **g** Effects of 1-MCP application on GL-3 and Ri-1 under greenhouse conditions. The data are the means ± SDs (*n* = 3 for **a**, three biological replicates; *n* = 12 for **b** and **d**, 12 biological replicates). **e** and **g** derived from two independent experiments with similar results, and one representative data point is shown. In comparison to GL-3, ***P < 0.001. The values not represented by the same letter are significantly different (*P* < 0.05). FW fresh weight. **e** Levels 1–4 indicate injury severity: (1) ≤1 leaf with necrosis on every plant; (2) 2–3 leaves; (3) 4–5 leaves; and (4) ≥6 leaves.

In addition to SA, jasmonic acid (JA) and ET can also regulate ROS-dependent PCD in plants^20^. Phytohormone analysis showed that the levels of both JA and JA-Ile were not significantly altered in Ri-1 compared with GL-3 (Table S3). However, the results of RNA-seq analysis revealed that DEGs were significantly enriched in response to ET (GO:0009723) in Ri-1 (Table S1). In addition, genes involved in ET biosynthesis and regulation were significantly upregulated in Ri-1, including *MdACO1* (MD10G1328100), *MdERF2* (MD07G1248400), and *MdMYC2-1* (MD06G1120000) (Table S2). RT-qPCR confirmed the upregulated levels of *MdACS4*, *MdACS5*, and *MdACO1* in Ri-2 (Fig. 4a).

Moreover, ET production increased in the RNAi lines on day 14 under normal and MI-depleted MS conditions. ET bursts were closely associated with necrosis in Ri-2 leaves (Figs. 4b and S3c). Increased ET production was observed only in RNAi lines and not in GL-3 and OE lines under MI-depleted conditions, suggesting the importance of the physiological MI level in fine-tuning the ET baseline in apple (Fig. 4b). Subsequently, two ET inhibitors, 1-methylcyclopropene (1-MCP), which prevents the binding of ET to its receptor and modulates tissue sensitivity to ET^39^, and cobalt dichloride (CoCl_2_), which inhibits ACO enzyme activity^40,41^, were applied. We observed that both ET inhibitors significantly suppressed necrosis development in the RNAi leaves (Fig. 4c–g). Thus, enhanced ET signaling promoted necrosis in RNAi apple lines.

### MI-mediated SA and ET signaling synergistically regulates senescence in apple

Our results revealed that 1-MCP treatment improved both SOD and anti-O_2_^−^ activity and reduced the accumulation of H_2_O_2_, and as well as levels of *MdSAG12*, *MdPR1*, and *MdPR5* in Ri-1. However, the role of 1-MCP was reversed when considering the SOD and anti-O_2_^−^ activity and *MdSAG12* expression in the GL-3 line (Fig. S6). This reversed role also led to slow growth in GL-3 (Fig. S7). Thus, it was suggested that activated ET signaling could induce ROS-induced PCD via the SA-dependent pathway in RNAi lines.

**Fig. 5:**
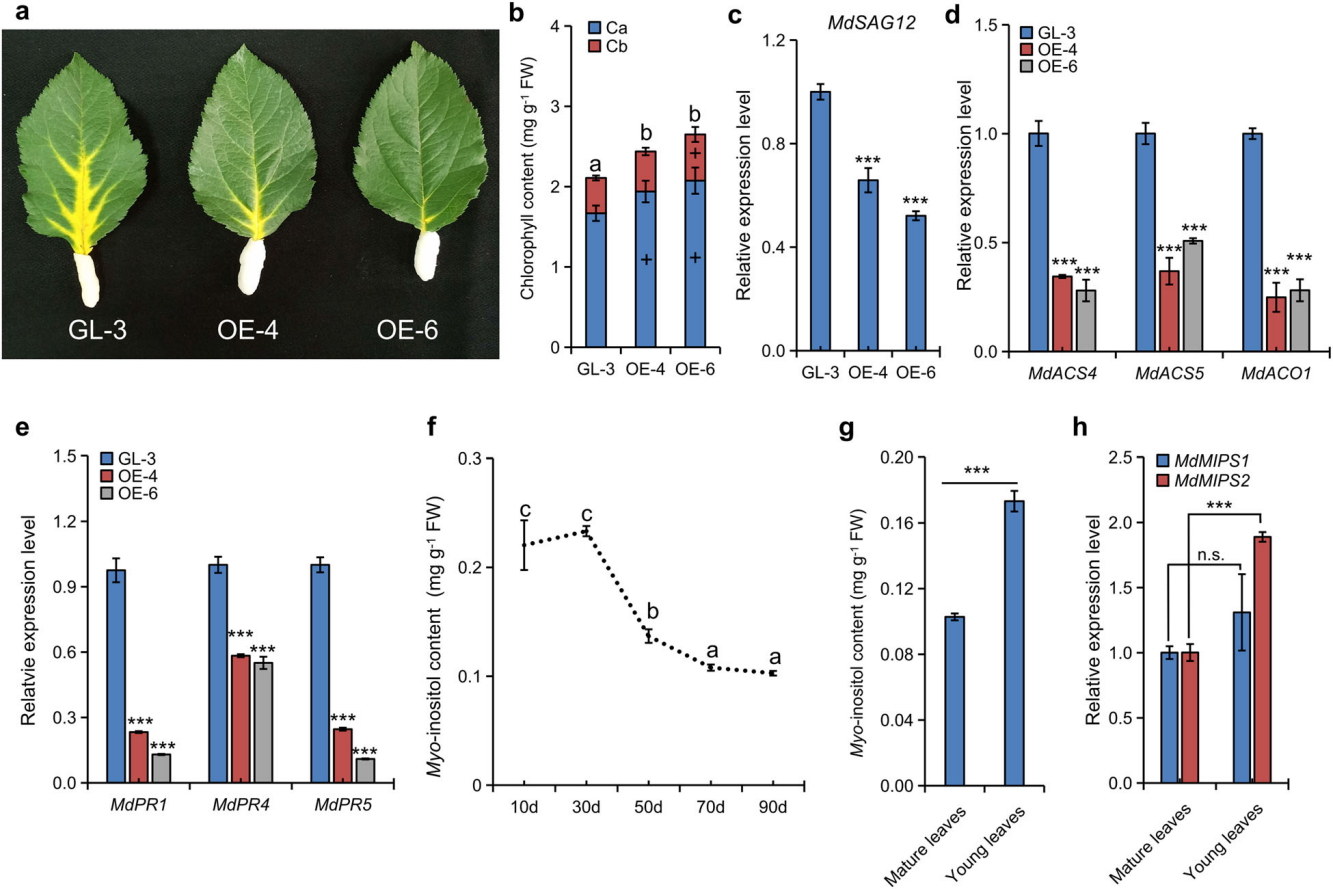
Involvement of *myo*-inositol in apple leaf senescence. **a** Delayed senescence in *MdMIPS1-*overexpressing apple leaves. Chlorophyll content **b** and expression levels of *MdSAG12* **c**, ET biosynthetic genes **d**, and *MdPR*s **e** in both GL-3 and *MdMIPS1-*overexpressing apple leaves of plants subjected to darkness-induced senescence. **f** MI levels in aging GL-3 leaves. MI levels **g** and *MdMIPS* expression levels **h** in both the young and mature leaves at the same time point. The data are the means ± SDs (*n* = 3, three biological replicates). In comparison to GL-3 or old leaves, ***P < 0.001; + indicates a significant increase (*P* < 0.05), and n.s. indicates no significant difference. The values not represented by the same letter are significantly different (*P* < 0.05). FW fresh weight.

An obvious delay in leaf senescence was found under dark conditions in the OE apple lines compared with GL-3 (Fig. 5a–c).

RT-qPCR showed that the levels of *MdPR*s, *MdACS*s, and *MdACO1* significantly decreased in OE apple leaves compared with the GL-3 leaves (Fig. 5d, e). In agreement with these findings, the MI level gradually decreased as the leaves of GL-3 aged (Fig. 5f). However, the levels of *MdSAG12*, ET biosynthetic genes, and *MdPR*s increased as the leaves aged; moreover, the MI level was low, and along with the decreased *MdMIPS2* expression level (Fig. S8). The MI level and *MdMIPS2* expression were higher in the young leaves than in the mature (older) leaves (90 days old) at the same time point (Fig. 5g, h). Thus, MI appears to have a major role in regulating senescence in apple. Taken together, these results indicated that activated SA and ET signaling synergistically induced rapid senescence in *MdMIPS1/2*-silenced lines.

## Discussion

MI is critical for plant biochemistry and physiology. In this study, we used *MdMIPS* transgenic apple lines to gain new insights into the physiological role of MI in apple. Our results showed that MI is vital to plant growth because it fine-tuned ROS-induced PCD through SA-dependent and ET-dependent pathways in apple.

In *A. thaliana*, the MIPS enzyme is encoded by three genes that encode biochemically similar proteins^14^. However, plants with mutations in these three genes show contrasting phenotypes, which was largely attributed to the differential expression patterns of the genes^14,15^. In apple, MIPS is likely encoded by *MdMIPS1* and *MdMIPS2*, and high similarities in both sequence and expression pattern suggest that these genes may be functionally equivalent. The most striking phenotypic feature of the MdMIPS1-RNAi lines was extensive PCD, which was manifested as autophagy and subsequent necrosis of the leaves and roots when plants were grown under MI-depleted conditions, regardless of the light intensity. MI complementation (or depletion) assays verified that the decreased MI production directly caused necrosis in *MdMIPS1/2*-silenced lines. Similarly, a lesion-like phenotype (cell death) was also noted in the *atmips1* mutant^15^ and AtMIPS cosuppression lines^42^. Sequence analysis showed a higher similarity between both MdMIPSs and AtMIPS3. AtMIPS3 has been reported to be essential for *Arabidopsis* embryo development^15^. Luo et al. found that a triple loss-of-function mutant was embryonically lethal^16^. By contrast, transgenic lines harboring severe cosuppression of *AtMIPS*s were characterized as having a phenotype similar to that of the *atmips1* mutant^42^. In addition, our results revealed normal meristems and leaf primordium when necrosis was apparent in *MdMIPS1/2-*silenced lines, demonstrating that decreased MI biosynthesis did not disrupt apple organogenesis; instead, it greatly perturbed plant growth.

The connection between MI biosynthesis and PCD has been reported to be SA-dependent in the *atmips1* mutant^14,15,43,44^. Here, we also observed constitutive SA production and activated SA signaling in *MdMIPS1*/2-silenced lines. Indeed, how MI modulates SA biosynthesis remains an open question. Donahue et al. reported elevated levels of ceramides and hydroxyceramides in the *atmips1* mutant, which may be a linker between the decreased biosynthesis of MI and increased SA production^14^. In this study, we revealed that the accumulation of WS pectin could stimulate SA accumulation in the MdMIPS1-RNAi lines by simulating the initial phase of pathogen ingress at the cell wall, as the cell wall was not degraded^36–38^. However, it is still unclear how decreased MI biosynthesis stimulates WS pectin accumulation in apple via the GlcA pathway^7^. Furthermore, enhanced SA signaling may induce ROS accumulation by interfering with SOD, POD, and anti-O_2_^−^ activities. It seems logical that a reduction in MI derivatives such as galactinol and raffinose-family oligosaccharides may contribute to the accumulation of ROS in *MdMIPS1/2*-silenced lines because these compounds have been reported to scavenge ROS^1^. Therefore, MdMIPS1-RNAi lines likely underwent spontaneous cell death due to a change in the sensitivity to oxidative stress induced by a compromised antioxidant system, which was closely associated with an elevated SA level resulting from an increase in WS pectin in cell wall polysaccharides.

We previously found that reduced MI biosynthesis may contribute to the accumulation of SA and ROS by stimulating WS pectin accumulation in MdUGT88F1-RNAi apple lines. However, relatively low levels of accumulated ROS may be in ineffective at promoting necrosis in MdUGT88F1-RNAi lines^45^. Moreover, our results revealed that ET bursts could accelerate necrosis in *MdMIPS1/2*-silenced lines. By contrast, the levels of ET biosynthetic genes were not altered in MdUGT88F1-RNAi lines (Table S4). Both ET biosynthesis and recognition have been reported to be required for active H_2_O_2_ production. High concentrations of both ET and ROS were observed to be temporally regulated in the same cells^20^. In addition, a functional ET pathway was demonstrated to be indispensable for the induction of an SA marker gene, and vice versa^46^. ET inhibitors consistently significantly alleviated ROS accumulation and repressed *MdPR* expression in the *MdMIPS1/2-*silenced lines. Even so, the enhanced production of ET may be mediated by an unknown MI derivative-dependent signaling pathway but not an SA-dependent signaling pathway in *MdMIPS1/2*-silenced lines. In addition, ET bursts may explain the phenotypic differences between MdMIPS1-RNAi lines and *atmips* mutants because previous studies failed to report enhanced ET production in *atmips* mutants^14–16,42–44^ or in other MIPS-suppressed plants^17,47^, along with the elevated SA level.

In addition, necrosis was largely restricted to parenchymal cells around the leaf vein, indicating that additional stress may have been a contributing factor by increasing transport pressure from leaves to sink tissues in *MdMIPS1/2*-silenced lines. Sorbitol is an example; in the *MdMIPS1/2-*silenced lines, an 78% increase in sorbitol caused not only transport pressure but also osmotic and oxidative stress to parenchymal cells around the leaf vein. The reduced ability to handle ROS increased the sensitivity to oxidative stress. Similar results were reported by Sheveleva et al.^48^. Consistent with these findings, increased sensitivity to sorbitol was also observed in the *atmips1* mutant^14^. Thus, the unique mechanism responsible for necrosis in *MdMIPS1/2*-silenced lines is logical.

Currently, it is controversial whether the accumulation of SA can improve biotic resistance in *atmips1* mutants^15,49^. Here, we did not observe a difference in the resistance of GL-3 and transgenic apple lines to *Valsa mali* (Fig. S9). ET signaling can activate resistance against necrotrophic pathogens and plays an opposite role in SA-mediated plant defense, for instance, against *Fusarium oxysporum* (*Fo*) in tomato^23,46,50^. Thus, enhanced ET and SA signaling may have antagonistically affected *Valsa* canker resistance in the *MdMIPS1/2*-silenced lines. However, further studies are needed to validate these results. In addition, during leaf senescence, the SA level gradually increases, which induces the expression of senescence-associated genes^23,24,51^. ET production has also been reported to increase in aging leaves^39^. Thus, it is likely that the activation of ET and SA signaling pathways can synergistically accelerate senescence in *MdMIPS1/2*-silenced lines. Indeed, RNA-seq analysis revealed strong enrichment in senescence-related processes in the MdMIPS1-RNAi lines, supported by the fact that the MI level decreased in the aging leaves. By contrast, an increased MI level in MdMIPS1-OE apple lines delayed leaf senescence. It seems that increased MI biosynthesis could compensate for the normal decrease in the MI level, thereby suppressing SA-mediated and ET-mediated signaling in MdMIPS1-OE apple lines during leaf senescence. In addition, it has been reported that ET causes leaf abscission by inhibiting auxin synthesis and transport or by enhancing auxin degradation^39^. Our results show that the auxin level significantly decreased in the MdMIPS1-RNAi leaves (Table S3). Thus, we argue that MdMIPS-mediated MI biosynthesis may be related to senescence in apple trees.

**Fig. 6:**
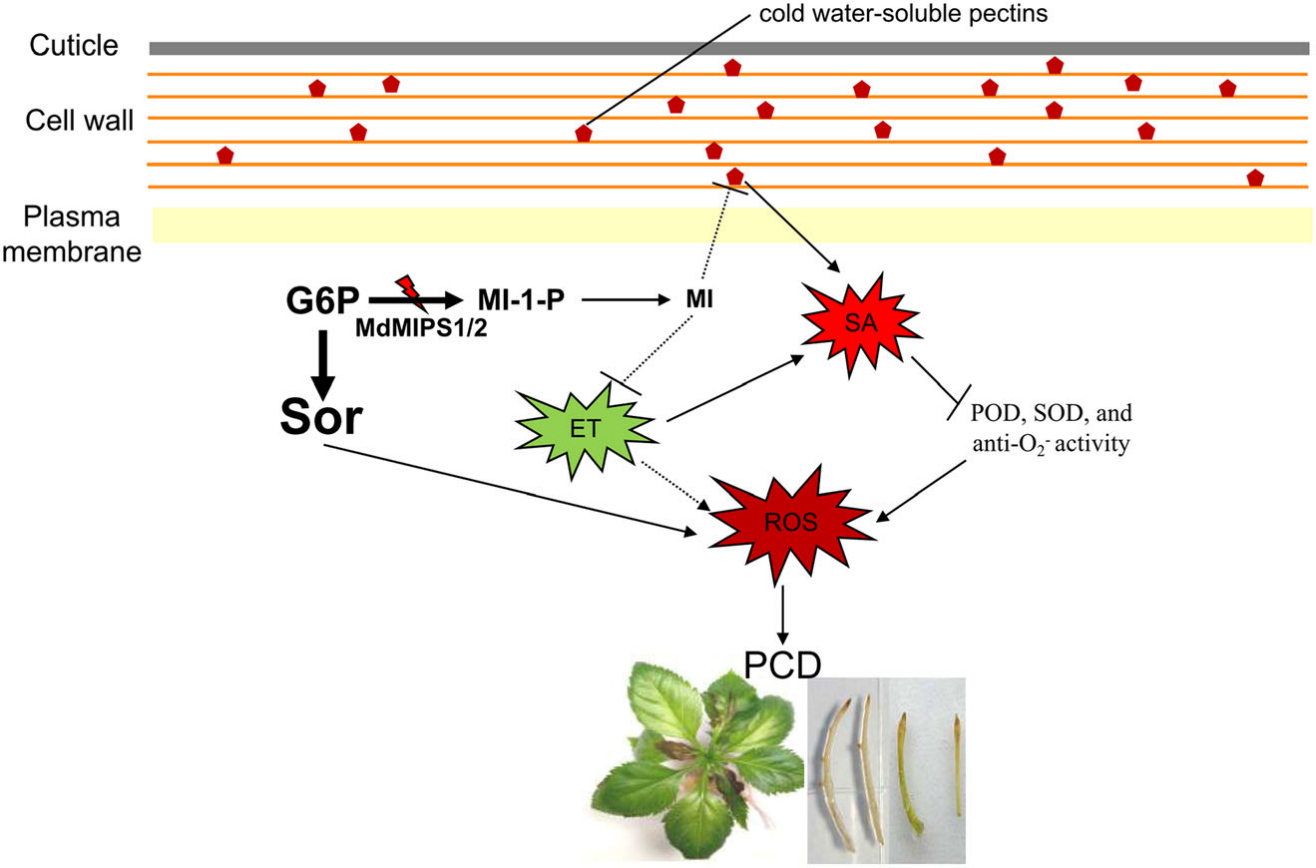
A model for *myo*-inositol regulation of ROS-induced PCD in apple. In *MdMIPS1/2-*silenced lines, decreased MI biosynthesis induces SA accumulation by stimulating the accumulation of WS pectin. Enhanced SA signaling directly compromises POD, SOD, and anti-O_2_^−^ activities, resulting in high ROS accumulation. Moreover, MI reduction also stimulates ET production and further induces ROS accumulation, possibly through SA-dependent and SA-independent pathways. Eventually, the accumulation of excess ROS directly leads to severe PCD and necrosis. In addition, metabolic influx of sorbitol causes additional oxidative stress and further accelerates necrosis around the leaf vein. G6P glucose-6-phosphate, Sor sorbitol, MI *myo*-inositol, and MI-1-P *myo*-inositol-1-phosphate. The solid and dashed lines refer to direct and indirect effects, respectively.

In conclusion, our results demonstrate that MI can regulate ROS-mediated PCD through SA-dependent and ET-dependent pathways (Fig. 6).

Decreasing MI biosynthesis in apple lines by RNA silencing of *MdMIPS1/2* caused extensive PCD, manifested as necrosis of both the leaves and roots and, ultimately, plant death once the supplemented MI was depleted. Excessive ROS accumulation contributed to necrosis, which was closely associated with the WS pectin-mediated increase in SA and the compromised antioxidant system. In addition, this ROS-induced PCD was accelerated by ET bursts. Moreover, the synergetic interplay between SA and ET was supported by delayed leaf senescence in MdMIPS1-OE apple lines. The metabolic influx of sorbitol may cause transport pressure and osmotic stress, thereby resulting in localized necrosis around the leaf veins in MdMIPS1-RNAi lines.

Necrosis was identified in both the roots and leaves of MdMIPS1-RNAi lines, which suggested a similar role of MI in apple roots and leaves. However, a limited investigation of root tissues was conducted here. This limitation may not perfectly reflect the physiological role of MI in apple roots. Roots are ultimately the organs that first and directly sense abiotic stresses, such as salinity, alkalinity, and drought. Thus, in the future, roots should be an organ of interest for the detailed testing of MdMIPS1-OE apple lines in terms of stress tolerance.

## Materials and methods

### Materials and growth conditions

Mature leaves, branches, bark, open flowers, and fruits were collected from healthy Royal Gala apple trees (Horticultural Experimental Station, Northwest A&F University, Yangling, China [34°20N, 108°24E]) at 9:00 a.m. and immediately frozen in liquid nitrogen before being stored at −80 °C.

GL-3, a line with a high regeneration capability and isolated from Royal Gala, was used for genetic transformation experiments^52^. GL-3 plants were subcultured every 4 weeks. After rooting on Murashige and Skoog (MS) agar media, transgenic and nontransgenic apple plantlets were transferred to small plastic pots (8 × 8 cm) containing a mixture of soil and perlite (1:1, v–v). After 10 days of acclimation in a growth chamber, the plants were transplanted to large plastic pots filled with soil and grown in a glasshouse. The plants were watered regularly and supplied with half-strength Hoagland’s nutrient solution (pH 6.0) once a week.

### Identification and phylogenetic analysis of *MdMIPS* genes in apple

To identify apple *MIPS* genes, we used *Arabidopsis thaliana* MIPS as a query sequence in the BLASTP program for searching the Rosaceae genome database (https://www.rosaceae.org/node/1), with an *E*-value cutoff of 0.01. Multiple sequence alignment and phylogenetic analysis were carried out as previously described^53^.

### Construction of plasmids and generation of transgenic apple plants

*MdMIPS1* was cloned from mature leaves of Royal Gala via reverse transcription-polymerase chain reaction (RT-PCR). To generate transgenic apple lines, the coding DNA region (CDS) of *MdMIPS1* was cloned and introduced into a pCAMBIA2300 vector to create an overexpression construct. pK7WIWG2D was used as an RNAi-mediated vector for silencing *MdMIPS1*. Thereafter, the *Agrobacterium*-mediated transformation of apple was carried out using the GL-3 line as the genetic background and the EHA105 strain^52^. The primers used for constructing all vectors are listed in Table S5.

### RT-qPCR analysis

RT-qPCR analysis was carried out as previously described^53^. The primers used for RT-qPCR are listed in Table S5.

### Assays of MI depletion and application of ET inhibitors

With respect to depletion assays, the main shoots of GL-3 and transgenic apple plantlets (after 4 weeks of subculturing) were cut into 1.5-cm segments, each of which included the first two leaves, and then rooted in MS agar media. After 12 days, the plants were transferred to a medium that lacked growth hormones to identify differences in performance upon exposure to normal media, MI-depleted MS media, and MI-depleted MS media consisting of 100 mM CoCl_2_ under long-day conditions (14 h/10 h [light/dark] photoperiod) at 23 °C. When necrosis was visible for most plants, the ET concentration was measured. The time of the first sign of necrosis was recorded.

After rooting on MS agar media, the GL-3 and Ri-1 plantlets were transplanted to a soil matrix. After 4 days, both GL-3 and Ri-1 plants were sealed in the same transparent chamber and exposed to 1-MCP (0.1 g/10 mL of water) daily under short-day conditions (10 h/14 h [light/dark] photoperiod; 22 °C/20 °C [day/night] temperature; 4000 lx illumination intensity). Control plants were exposed to water only. After treatment with 1-MCP for 18 days, the plants were imaged, followed by measurements of the injury index and various growth parameters. The aerial parts of plants (with no visible lesions) treated for 10 days were collected, immediately frozen in liquid nitrogen, and then stored at −80 °C for subsequent analyses of enzymatic activity, H_2_O_2_ concentrations, and gene expression.

### Microscopy analysis

To examine the cells, tissues were excised from the stem apexes, leaves, and roots and stained with toluidine blue O as previously described^45^.

To examine the cell walls and other organelles, as described by Sun et al.^54^, TEM analysis was performed.

### Assays of antioxidant enzyme activities and determination of ROS accumulation

SOD, POD, and anti-O_2_^−^ activities, as well as H_2_O_2_ levels, were determined by using detection kits (Nanjing Jiancheng Bioengineering Institute, China) according to the manufacturer’s instructions. ~0.1 g (FW) of fine powder was used for each sample. Total protein was determined using Coomassie brilliant blue staining according to the methods of Bradford^55^. The accumulations of H_2_O_2_ and O_2_^−^ were evaluated by histochemical staining methods that used diaminobenzidine (DAB) and nitro blue tetrazolium (NBT), respectively^32^. Detection of cell death using trypan blue staining was performed as described by Dahro et al.^56^.

### Determination of lignin and cell wall polysaccharide concentrations

Samples of GL-3 and transgenic apple lines were dried at 45 °C for 14 days and then ground to a fine powder. The concentrations of lignin, hemicellulose, and cellulose were determined using the Van Soest method^57^. Pectin was extracted and measured as described by Gallego-Giraldo et al.^37^.

### Metabolome analysis

The aerial parts of 15 days old GL-3 and Ri-1 (with no visible lesions) were collected, frozen immediately in liquid nitrogen, and then stored at −80 °C. The samples were then transported to Wuhan MetWare Biotechnology Co., Ltd., China, for analysis of the metabolome and phytohormone levels^58^. Three biological replicates were included, and each biological replication included at least 10 plants.

The soluble sugars and MI were quantified as previously described^32^, and the SA measurements were performed as previously described^45^.

ET production was measured according to the methods of Zhu et al.^59^, with minor modifications. Briefly, two apple plantlets were rooted on MS agar media in a 0.25-L airtight tissue culture vessel for treatment, and a 5-mL gas sample was collected with a syringe. The ET level was then determined using a GC-14A gas chromatograph (Shimadzu, Japan) fitted with a flame ionization detector (FID) and an activated alumina column (200 × 0.3 cm). The oven, detector, and injector were operated at 70, 70, and 150 °C, respectively. The carrier gas (N_2_, H_2_, and air) flow rates were 30, 30, and 300 mL min^−1^, respectively. ET production was expressed as microliters per hour per kilogram of fresh weight (FW).

### RNA sequencing

The plant materials used for RNA-seq analysis were identical to those used in the metabolome and phytohormone analyses. RNA-seq analysis was performed as previously described^45^.

### Assays of pathogen infection and leaf senescence

Assays of *Valsa mali* infection were performed as described by Zhou et al.^45^. Fully mature leaves of 6 months old GL-3 and MdMIPS1-OE plants were subjected to darkness-induced senescence as described by Wang et al.^60^.

### Statistical analysis

SPSS (version 17.0) was used for the statistical analysis. The data were subjected to one-way ANOVA and are reported as the means ± standard deviations (SDs).

## Supplementary information

Supplementary information

## References

[1] Valluru R, Van den Ende W (2011). *Myo*-inositol and beyond—emerging networks under stress. Plant Sci..

[2] Van den Ende W (2013). Multifunctional fructans and raffinose family oligosaccharides. Front. Plant Sci..

[3] Taji T, Takahashi S, Shinozaki K (2006). Inositols and their metabolites in abiotic and biotic stress responses. Subcell. Biochem..

[4] Zhai H (2016). A *myo*-inositol-1-phosphate synthase gene, *IbMIPS1*, enhances salt and drought tolerance and stem nematode resistance in transgenic sweet potato. Plant Biotechnol. J..

[5] Wang W (2008). An inositolphosphorylceramide synthase is involved in regulation of plant programmed cell death associated with defense in *Arabidopsis*. Plant Cell.

[6] Berkey R, Bendigeri D, Xiao S (2012). Sphingolipids and plant defense/disease: the “death” connection and beyond. Front. Plant Sci..

[7] Loewus FA (2006). Inositol and plant cell wall polysaccharide biogenesis. Subcell. Biochem..

[8] Loewus FA, Murthy PPN (2000). *Myo*-Inositol metabolism in plants. Plant Sci..

[9] Eisenberg F, Bolden AH, Loewus FA (1964). Inositol formation by cyclization of glucose chain in rat testis. Biochem. Biophys. Res. Commun..

[10] Majumder AL, Chatterjee A, Ghosh Dastidar K, Majee M (2003). Diversification and evolution of L-*myo*-inositol 1-phosphate synthase. FEBS Lett..

[11] Hazra A, Dasgupta N, Sengupta S, Das S (2019). MIPS: Functional dynamics in evolutionary pathways of plant kingdom. Genomics.

[12] Ghosh Dastidar K, Chatterjee A, Chatterjee A, Majumder AL (2006). Evolutionary divergence of l-*myo*-inositol 1-phosphate synthase: significance of a “core catalytic structure”. Subcell. Biochem..

[13] Torabinejad J, Gillaspy GE (2006). Functional genomics of inositol metabolism. Subcell. Biochem..

[14] Donahue JL (2010). The *Arabidopsis thaliana myo*-Inositol 1-phosphate synthase1 gene is required for *myo*-inositol synthesis and suppression of cell death. Plant Cell.

[15] Meng PH (2009). Crosstalks between *myo*-inositol metabolism, programmed cell death and basal immunity in *Arabidopsis*. PLoS ONE.

[16] Luo Y (2011). d-*myo*-inositol-3-phosphate affects phosphatidylinositol-mediated endomembrane function in *Arabidopsis* and is essential for auxin-regulated embryogenesis. Plant Cell.

[17] Keller R, Brearley CA, Trethewey RN, Müller-Röber B (1998). Reduced inositol content and altered morphology in transgenic potato plants inhibited for 1D-*myo*-inositol-3-phosphate synthase. Plant J..

[18] van Doorn WG, Woltering EJ (2004). Senescence and programmed cell death: substance or semantics?. J. Exp. Bot..

[19] Williams B, Dickman M (2008). Plant programmed cell death: can’t live with it; can’t live without it. Mol. Plant Pathol..

[20] Overmyer K, Brosché M, Kangasjärvi J (2003). Reactive oxygen species and hormonal control of cell death. Trends Plant Sci..

[21] Malik, B. et al. Plant signaling: response to reactive oxygen species In *Plant Signaling: Understanding the Molecular Crosstalk* 1–38 (Springer, New Delhi, 2014).

[22] Sharma P, Jha AB, Dubey RS, Pessarakli M (2012). Reactive oxgen species, oxidative damage, and antioxidative defense mechanism in plants under stressful conditions. J. Bot..

[23] Vlot AC, Dempsey DA, Klessig DF (2009). Salicylic acid, a multifaceted hormone to combat disease. Annu. Rev. Phytopathol..

[24] Guo P (2017). A tripartite amplification loop involving the transcription factor WRKY75, salicylic acid, and reactive oxygen species accelerates leaf senescence. Plant Cell.

[25] Adams-Phillips L, Barry C, Giovannoni J (2004). Signal transduction systems regulating fruit ripening. Trends Plant Sci..

[26] Xu J, Zhang S (2014). Regulation of ethylene biosynthesis and signaling by protein kinases and phosphatases. Mol. Plant.

[27] Fluhr R, Mattoo AK, Dilley DR (1996). Ethylene-biosynthesis and perception. Crit. Rev. Plant Sci..

[28] Cin VD, Danesin M, Boschetti A, Dorigoni A, Ramina A (2005). Ethylene biosynthesis and perception in apple fruitlet abscission (*Malus domestica* L. Borck). J. Exp. Bot..

[29] Joo S, Liu Y, Lueth A, Zhang S (2008). MAPK phosphorylation-induced stabilization of ACS6 protein is mediated by the non-catalytic C-terminal domain, which also contains the cis-determinant for rapid degradation by the 26S proteasome pathway. Plant J..

[30] Christians MJ (2009). The BTB ubiquitin ligases ETO1, EOL1 and EOL2 act collectively to regulate ethylene biosynthesis in *Arabidopsis* by controlling type-2 ACC synthase levels. Plant J..

[31] Xu H (2018). Transcriptome analysis reveals a regulation of ethylene-induced post-harvest senescence in pear fruit. Sci. Hortic..

[32] Hu L (2018). Exogenous *myo*-inositol alleviates salinity-induced stress in *Malus hupehensis* Rehd. Plant Physiol. Biochem..

[33] Velasco R (2010). The genome of the domesticated apple (*Malus × domestica* Borkh.). Nat. Genet..

[34] Basak P (2017). An evolutionary analysis identifies a conserved pentapeptide stretch containing the two essential lysine residues for rice l-myo-inositol 1-phosphate synthase catalytic activity. PLoS ONE.

[35] Yu SW (1999). Cellular and genetic responses of plants to sugar starvation. Plant Physiol..

[36] Vorwerk S, Somerville S, Somerville C (2004). The role of plant cell wall polysaccharide composition in disease resistance. Trends Plant Sci..

[37] Gallego-Giraldo L, Jikumaru Y, Kamiya Y, Tang Y, Dixon RA (2011). Selective lignin downregulation leads to constitutive defense response expression in alfalfa (*Medicago sativa* L.). N. Phytol..

[38] Gallego-Giraldo L, Escamilla-Trevino L, Jackson L, Dixon RA (2011). Salicylic acid mediates the reduced growth of lignin down-regulated plants. Proc. Natl Acad. Sci. USA.

[39] Iqbal N (2017). Ethylene role in plant growth, development and senescence: interaction with other phytohormones. Front. Plant Sci..

[40] Yu YB, Yang SF (1979). Auxin-induced ethylene production and its inhibition by aminoethoxyvinyiglycine and cobalt ion. Plant Physiol..

[41] Merritt F, Kemper A, Tallman G (2001). Inhibitors of ethylene synthesis inhibit auxin-induced stomatal opening in epidermis detached from leaves of *Vicia faba* L. Plant Cell Physiol..

[42] Fleet CM, Yen JY, Hill EA, Gillaspy GE (2018). Co-suppression of AtMIPS demonstrates cooperation of MIPS1, MIPS2 and MIPS3 in maintaining *myo*-inositol synthesis. Plant Mol. Biol..

[43] Bruggeman Q (2015). Involvement of *Arabidopsis* hexokinase1 in cell death mediated by *myo*-inositol accumulation. Plant Cell.

[44] Ma L (2016). *Arabidopsis* FHY3 and FAR1 regulate light-induced *myo*-inositol biosynthesis and oxidative stress responses by transcriptional activation of MIPS1. Mol. Plant.

[45] Zhou K (2019). MdUGT88F1-mediated phloridzin biosynthesis regulates apple development and *Valsa* canker resistance. Plant Physiol..

[46] Di X, Comila J, Takken FLW (2017). Involvement of salicylic acid, ethylene and jasmonic acid signalling pathways in the susceptibility of tomato to *Fusarium oxysporum*. Mol. Plant Pathol..

[47] Nunes AC (2006). RNAi-mediated silencing of the *myo*-inositol-1-phosphate synthase gene (*GmMIPS1*) in transgenic soybean inhibited seed development and reduced phytate content. Planta.

[48] Sheveleva EV (1998). Sorbitol-6-phosphate dehydrogenase expression in transgenic tobacco. High amounts of sorbitol lead to necrotic lesions. Plant Physiol..

[49] Murphy AM, Otto B, Brearley CA, Carr JP, Hanke DE (2008). A role for inositol hexakisphosphate in the maintenance of basal resistance to plant pathogens. Plant J..

[50] Glazebrook J (2005). Contrasting mechanisms of defense against biotrophic and necrotrophic pathogens. Annu. Rev. Phytopathol..

[51] Gepstein S (2003). Large-scale identification of leaf senescence-associated genes. Plant J..

[52] Dai H (2013). Development of a seedling clone with high regeneration capacity and susceptibility to *Agrobacterium* in apple. Sci. Hortic..

[53] Zhou K, Hu L, Li P, Gong X, Ma F (2017). Genome-wide identification of glycosyltransferases converting phloretin to phloridzin in *Malus* species. Plant Sci..

[54] Sun X (2018). Improvement of drought tolerance by overexpressing *MdATG18a* is mediated by modified antioxidant system and activated autophagy in transgenic apple. Plant Biotechnol. J..

[55] Bradford MM (1976). A rapid and sensitive method for the quantitation of microgram quantities of protein utilizing the principle of protein–dye binding. Anal. Biochem..

[56] Dahro B, Wang F, Peng T, Liu JH (2016). *PtrA/NINV*, an alkaline/neutral invertase gene of *Poncirus trifoliata*, confers enhanced tolerance to multiple abiotic stresses by modulating ROS levels and maintaining photosynthetic efficiency. BMC Plant Biol..

[57] Van Soest PJ (1963). The use of detergents in the analysis of fibrous feeds: II. A rapid method for the determination of fiber and lignin. J. Assoc. Off. Anal. Chem..

[58] Chen W (2013). Novel integrated method for large-scale detection, identification, and quantification of widely targeted metabolites: application in the study of rice metabolomics. Mol. Plant..

[59] Zhu Q (2013). Identification of xyloglucan endotransglucosylase/hydrolase genes (*XTHs*) and their expression in persimmon fruit as influenced by 1-methylcyclopropene and gibberellic acid during storage at ambient temperature. Food Chem..

[60] Wang P (2012). Delayed senescence of apple leaves by exogenous melatonin treatment: toward regulating the ascorbate-glutathione cycle. J. Pineal Res..

