# Supplementary material for: Myo-inositol mediates reactive oxygen species-induced programmed cell death via salicylic acid-dependent and ethylene-dependent pathways in apple
Source: Hortic Res. 2020 Sep 1;7:138. doi: 10.1038/s41438-020-00357-2 (PMC7459343; doi:10.1038/s41438-020-00357-2)
Supplement: Supplementary file 1 — Supplementary information [file 41438_2020_357_MOESM1_ESM.doc]

**Table S1 List of Gene Ontology terms statistically enriched in differentially-expressed genes (DEGs) of Ri-1 by RNA-seq analysis (p. adjust < 0.05).**

| **ID** | **Description** | **p.adjust** | **up** | **down** |
| --- | --- | --- | --- | --- |
| GO:0019336 | phenol-containing compound catabolic process | 4.47294E-12 | 20 | 0 |
| GO:0044550 | secondary metabolite biosynthetic process | 3.74268E-11 | 58 | 10 |
| GO:0018958 | phenol-containing compound metabolic process | 6.54251E-10 | 29 | 0 |
| GO:0007568 | aging | 1.02794E-08 | 48 | 5 |
| GO:0010150 | leaf senescence | 4.26188E-08 | 42 | 4 |
| GO:0046244 | salicylic acid catabolic process | 4.26188E-08 | 14 | 0 |
| GO:0090693 | plant organ senescence | 6.18794E-08 | 42 | 4 |
| GO:0017001 | antibiotic catabolic process | 1.72359E-07 | 26 | 7 |
| GO:0042737 | drug catabolic process | 2.085E-07 | 41 | 11 |
| GO:0010243 | response to organonitrogen compound | 2.67715E-07 | 46 | 9 |
| GO:1901698 | response to nitrogen compound | 1.13074E-06 | 49 | 12 |
| GO:0009696 | salicylic acid metabolic process | 1.13074E-06 | 20 | 0 |
| GO:0016999 | antibiotic metabolic process | 3.25803E-06 | 40 | 8 |
| GO:0010200 | response to chitin | 3.25803E-06 | 26 | 8 |
| GO:0002239 | response to oomycetes | 4.20593E-06 | 28 | 3 |
| GO:0002229 | defense response to oomycetes | 4.35937E-06 | 25 | 3 |
| GO:1901136 | carbohydrate derivative catabolic process | 5.25049E-06 | 18 | 2 |
| GO:1901616 | organic hydroxy compound catabolic process | 5.25049E-06 | 20 | 0 |
| GO:0042537 | benzene-containing compound metabolic process | 3.46116E-05 | 20 | 0 |
| GO:0051055 | negative regulation of lipid biosynthetic process | 4.94823E-05 | 4 | 4 |
| GO:0006884 | cell volume homeostasis | 9.29555E-05 | 7 | 0 |
| GO:0042447 | hormone catabolic process | 0.000105402 | 14 | 0 |
| GO:0009751 | response to salicylic acid | 0.000115979 | 44 | 6 |
| GO:0045833 | negative regulation of lipid metabolic process | 0.000145549 | 4 | 4 |
| GO:0052314 | phytoalexin metabolic process | 0.000145549 | 8 | 0 |
| GO:0052315 | phytoalexin biosynthetic process | 0.000145549 | 8 | 0 |
| GO:0071417 | cellular response to organonitrogen compound | 0.000184007 | 16 | 1 |
| GO:1900055 | regulation of leaf senescence | 0.000305267 | 15 | 1 |
| GO:0006816 | calcium ion transport | 0.000359921 | 15 | 1 |
| GO:1901699 | cellular response to nitrogen compound | 0.000388264 | 18 | 4 |
| GO:0009404 | toxin metabolic process | 0.000424416 | 13 | 1 |
| GO:0030001 | metal ion transport | 0.000672504 | 37 | 11 |
| GO:0071230 | cellular response to amino acid stimulus | 0.000672504 | 12 | 1 |
| GO:1900056 | negative regulation of leaf senescence | 0.000698954 | 9 | 1 |
| GO:1905622 | negative regulation of leaf development | 0.000932048 | 9 | 1 |
| GO:0042445 | hormone metabolic process | 0.001124423 | 46 | 6 |
| GO:0009699 | phenylpropanoid biosynthetic process | 0.001533672 | 27 | 5 |
| GO:1901615 | organic hydroxy compound metabolic process | 0.001788269 | 47 | 4 |
| GO:0044273 | sulfur compound catabolic process | 0.00191332 | 10 | 0 |
| GO:0010120 | camalexin biosynthetic process | 0.00191332 | 6 | 0 |
| GO:0052317 | camalexin metabolic process | 0.00191332 | 6 | 0 |
| GO:0080187 | floral organ senescence | 0.001956728 | 4 | 3 |
| GO:0071407 | cellular response to organic cyclic compound | 0.002698035 | 28 | 11 |
| GO:0009814 | defense response, incompatible interaction | 0.002973673 | 35 | 7 |
| GO:0009700 | indole phytoalexin biosynthetic process | 0.002998032 | 6 | 0 |
| GO:0046217 | indole phytoalexin metabolic process | 0.002998032 | 6 | 0 |
| GO:0009698 | phenylpropanoid metabolic process | 0.003149006 | 30 | 7 |
| GO:0070838 | divalent metal ion transport | 0.003466208 | 18 | 1 |
| GO:0043200 | response to amino acid | 0.003466208 | 12 | 1 |
| GO:0048584 | positive regulation of response to stimulus | 0.003711452 | 39 | 14 |
| GO:0072511 | divalent inorganic cation transport | 0.003743328 | 18 | 1 |
| GO:0019722 | calcium-mediated signaling | 0.004322435 | 12 | 2 |
| GO:0016106 | sesquiterpenoid biosynthetic process | 0.004677406 | 11 | 1 |
| GO:0009787 | regulation of abscisic acid-activated signaling pathway | 0.004822959 | 26 | 3 |
| GO:1901419 | regulation of response to alcohol | 0.004822959 | 26 | 3 |
| GO:1905957 | regulation of cellular response to alcohol | 0.004822959 | 26 | 3 |
| GO:0010623 | programmed cell death involved in cell development | 0.004822959 | 8 | 0 |
| GO:0048544 | recognition of pollen | 0.005928981 | 27 | 1 |
| GO:0009403 | toxin biosynthetic process | 0.005928981 | 8 | 0 |
| GO:0019932 | second-messenger-mediated signaling | 0.006052676 | 13 | 2 |
| GO:0071215 | cellular response to abscisic acid stimulus | 0.006804214 | 39 | 13 |
| GO:0097306 | cellular response to alcohol | 0.006804214 | 39 | 13 |
| GO:0072329 | monocarboxylic acid catabolic process | 0.007328496 | 18 | 0 |
| GO:0008037 | cell recognition | 0.007365816 | 27 | 1 |
| GO:0042743 | hydrogen peroxide metabolic process | 0.008591897 | 17 | 7 |
| GO:0052542 | defense response by callose deposition | 0.008782819 | 9 | 0 |
| GO:0007166 | cell surface receptor signaling pathway | 0.009819386 | 30 | 8 |
| GO:0009875 | pollen-pistil interaction | 0.009819386 | 28 | 1 |
| GO:0009741 | response to brassinosteroid | 0.009819386 | 14 | 11 |
| GO:0009788 | negative regulation of abscisic acid-activated signaling pathway | 0.009819386 | 17 | 0 |
| GO:1901420 | negative regulation of response to alcohol | 0.009819386 | 17 | 0 |
| GO:1905958 | negative regulation of cellular response to alcohol | 0.009819386 | 17 | 0 |
| GO:0043902 | positive regulation of multi-organism process | 0.01033826 | 13 | 3 |
| GO:0062014 | negative regulation of small molecule metabolic process | 0.010523268 | 4 | 4 |
| GO:0042744 | hydrogen peroxide catabolic process | 0.01059545 | 12 | 7 |
| GO:0009566 | fertilization | 0.010680093 | 10 | 2 |
| GO:0009738 | abscisic acid-activated signaling pathway | 0.010950928 | 37 | 11 |
| GO:0009809 | lignin biosynthetic process | 0.012113552 | 16 | 4 |
| GO:0030104 | water homeostasis | 0.012536561 | 7 | 0 |
| GO:0009813 | flavonoid biosynthetic process | 0.015702881 | 18 | 8 |
| GO:0009808 | lignin metabolic process | 0.016850289 | 18 | 6 |
| GO:0009812 | flavonoid metabolic process | 0.017663945 | 21 | 8 |
| GO:1900426 | positive regulation of defense response to bacterium | 0.017663945 | 9 | 3 |
| GO:0008361 | regulation of cell size | 0.018586399 | 8 | 2 |
| GO:0010148 | transpiration | 0.01859497 | 2 | 3 |
| GO:0010648 | negative regulation of cell communication | 0.019058591 | 21 | 2 |
| GO:0023057 | negative regulation of signaling | 0.019058591 | 21 | 2 |
| GO:0006714 | sesquiterpenoid metabolic process | 0.019058591 | 11 | 1 |
| GO:0045926 | negative regulation of growth | 0.019058591 | 9 | 2 |
| GO:0006561 | proline biosynthetic process | 0.019058591 | 6 | 1 |
| GO:0010029 | regulation of seed germination | 0.019408515 | 12 | 6 |
| GO:0009688 | abscisic acid biosynthetic process | 0.020548417 | 8 | 1 |
| GO:0043289 | apocarotenoid biosynthetic process | 0.020548417 | 8 | 1 |
| GO:1902645 | tertiary alcohol biosynthetic process | 0.020548417 | 8 | 1 |
| GO:0006560 | proline metabolic process | 0.020548417 | 7 | 1 |
| GO:1902288 | regulation of defense response to oomycetes | 0.024299805 | 7 | 2 |
| GO:0030308 | negative regulation of cell growth | 0.024377323 | 7 | 1 |
| GO:0009723 | response to ethylene | 0.027617583 | 36 | 11 |
| GO:0019953 | sexual reproduction | 0.031980881 | 17 | 8 |
| GO:0016054 | organic acid catabolic process | 0.032110748 | 23 | 2 |
| GO:0046395 | carboxylic acid catabolic process | 0.032110748 | 23 | 2 |
| GO:0009311 | oligosaccharide metabolic process | 0.032110748 | 17 | 2 |
| GO:0006026 | aminoglycan catabolic process | 0.032110748 | 8 | 0 |
| GO:0006030 | chitin metabolic process | 0.032110748 | 8 | 0 |
| GO:0006032 | chitin catabolic process | 0.032110748 | 8 | 0 |
| GO:0046348 | amino sugar catabolic process | 0.032110748 | 8 | 0 |
| GO:1901072 | glucosamine-containing compound catabolic process | 0.032110748 | 8 | 0 |
| GO:0097501 | stress response to metal ion | 0.032110748 | 5 | 0 |
| GO:0009968 | negative regulation of signal transduction | 0.033039055 | 21 | 1 |
| GO:1900140 | regulation of seedling development | 0.033039055 | 12 | 6 |
| GO:0072523 | purine-containing compound catabolic process | 0.037662839 | 7 | 0 |
| GO:0080167 | response to karrikin | 0.039573734 | 14 | 5 |
| GO:0042391 | regulation of membrane potential | 0.039573734 | 12 | 2 |
| GO:0072529 | pyrimidine-containing compound catabolic process | 0.039573734 | 2 | 3 |
| GO:0002215 | defense response to nematode | 0.039573734 | 2 | 2 |
| GO:0035066 | positive regulation of histone acetylation | 0.039573734 | 0 | 4 |
| GO:1901985 | positive regulation of protein acetylation | 0.039573734 | 0 | 4 |
| GO:2000758 | positive regulation of peptidyl-lysine acetylation | 0.039573734 | 0 | 4 |
| GO:0007338 | single fertilization | 0.041529363 | 8 | 0 |
| GO:1901071 | glucosamine-containing compound metabolic process | 0.048685366 | 8 | 0 |
| GO:0044421 | extracellular region part | 3.3161E-08 | 21 | 16 |
| GO:0048046 | apoplast | 5.02348E-07 | 27 | 41 |
| GO:0031225 | anchored component of membrane | 3.0604E-06 | 22 | 26 |
| GO:0005615 | extracellular space | 4.29981E-06 | 16 | 14 |
| GO:0090406 | pollen tube | 4.29981E-06 | 19 | 2 |
| GO:0120025 | plasma membrane bounded cell projection | 6.66954E-05 | 23 | 3 |
| GO:0042995 | cell projection | 0.000233824 | 23 | 3 |
| GO:0005788 | endoplasmic reticulum lumen | 0.000277668 | 16 | 0 |
| GO:0046658 | anchored component of plasma membrane | 0.00052691 | 12 | 21 |
| GO:0031012 | extracellular matrix | 0.001919072 | 5 | 2 |
| GO:0044815 | DNA packaging complex | 0.00389538 | 0 | 17 |
| GO:0000786 | nucleosome | 0.00389538 | 0 | 16 |
| GO:0010282 | senescence-associated vacuole | 0.00454456 | 7 | 0 |
| GO:0032993 | protein-DNA complex | 0.0047635 | 0 | 19 |
| GO:0043680 | filiform apparatus | 0.009882843 | 7 | 0 |
| GO:0009505 | plant-type cell wall | 0.010096037 | 12 | 26 |
| GO:0005764 | lysosome | 0.013706997 | 11 | 2 |
| GO:0000323 | lytic vacuole | 0.026832053 | 11 | 2 |
| GO:0001871 | pattern binding | 3.12486E-06 | 29 | 6 |
| GO:0030247 | polysaccharide binding | 3.12486E-06 | 29 | 6 |
| GO:0004497 | monooxygenase activity | 4.61045E-05 | 50 | 11 |
| GO:0005506 | iron ion binding | 0.000596894 | 49 | 9 |
| GO:0016709 | oxidoreductase activity, acting on paired donors, with incorporation or reduction of molecular oxygen, NAD(P)H as one donor, and incorporation of one atom of oxygen | 0.000596894 | 27 | 6 |
| GO:0004970 | ionotropic glutamate receptor activity | 0.000596894 | 12 | 1 |
| GO:0005230 | extracellular ligand-gated ion channel activity | 0.000596894 | 12 | 1 |
| GO:0008066 | glutamate receptor activity | 0.000596894 | 12 | 1 |
| GO:0022824 | transmitter-gated ion channel activity | 0.000596894 | 12 | 1 |
| GO:0022835 | transmitter-gated channel activity | 0.000596894 | 12 | 1 |
| GO:0030594 | neurotransmitter receptor activity | 0.000596894 | 12 | 1 |
| GO:0015276 | ligand-gated ion channel activity | 0.001278458 | 13 | 1 |
| GO:0022834 | ligand-gated channel activity | 0.001278458 | 13 | 1 |
| GO:0030551 | cyclic nucleotide binding | 0.001901558 | 12 | 2 |
| GO:0030552 | cAMP binding | 0.001901558 | 12 | 2 |
| GO:0030553 | cGMP binding | 0.001901558 | 12 | 2 |
| GO:0008083 | growth factor activity | 0.004218581 | 5 | 1 |
| GO:0004364 | glutathione transferase activity | 0.005473132 | 15 | 1 |
| GO:0019840 | isoprenoid binding | 0.005508023 | 2 | 5 |
| GO:0005262 | calcium channel activity | 0.006257398 | 13 | 1 |
| GO:0008378 | galactosyltransferase activity | 0.008165264 | 12 | 2 |
| GO:0008422 | beta-glucosidase activity | 0.008254318 | 16 | 7 |
| GO:0022838 | substrate-specific channel activity | 0.013492885 | 25 | 6 |
| GO:0042973 | glucan endo-1,3-beta-D-glucosidase activity | 0.013492885 | 10 | 5 |
| GO:0005216 | ion channel activity | 0.026440055 | 24 | 3 |
| GO:0004568 | chitinase activity | 0.027009688 | 8 | 0 |
| GO:0010427 | abscisic acid binding | 0.041145321 | 0 | 5 |
| GO:0015926 | glucosidase activity | 0.044251116 | 17 | 7 |
| GO:0015267 | channel activity | 0.048246378 | 26 | 7 |
| GO:0022803 | passive transmembrane transporter activity | 0.048246378 | 26 | 7 |
| GO:0051213 | dioxygenase activity | 0.048246378 | 29 | 2 |
| GO:0015085 | calcium ion transmembrane transporter activity | 0.048345989 | 18 | 1 |

**Table S2 Differentially-expressed genes involved in SA- and ET-related processes in Ri-1 compared with GL-3 by RNA-seq analysis.**

| Gene ID | Gene description | Ri-1/GL-3 | |
| --- | --- | --- | --- |
|  |  | log2Fold Change | padj |
| MD05G1109100 | PR1; pathogenesis-related protein 1-like | 2.3 | 9.61276E-08 |
| MD14G1080100 | PR2;  glucan endo-1,3-beta-glucosidase | 3.317 | 2.57945E-18 |
| MD14G1080000 |  | 3.755 | 2.43025E-05 |
| MD14G1079900 |  | 3.246 | 4.23978E-13 |
| MD02G1120300 | PR3-1; endochitinase-like | 1.175 | 0.001400661 |
| MD01G1213300 | PR3-2; acidic endochitinase-like | 2.417 | 9.73637E-05 |
| MD01G1212900 |  | 5.954 | 3.4914E-05 |
| MD01G1213100 |  | 2.564 | 0.000414677 |
| MD02G1120300 | PR4; endochitinase-like | 1.175 | 0.001400661 |
| MD01G1213300 | PR8; acidic endochitinase-like | 2.417 | 9.73637E-05 |
| MD01G1213100 |  | 2.564 | 0.000414677 |
| MD01G1212900 |  | 5.954 | 3.4914E-05 |
| MD13G1160700 | PR10; Mal d 1-like | 2.469 | 7.48836E-13 |
| MD13G1160500 |  | 2.063 | 2.00352E-60 |
| MD13G1160500 |  | 4.107 | 1.53252E-06 |
| MD13G1161200 |  | 2.432 | 1.91407E-08 |
| MD16G1160000 |  | 1.811 | 1.19913E-14 |
| MD13G1161100 |  | 2.095 | 7.1993E-10 |
| MD13G1160900 |  | 2.275 | 2.6459E-09 |
| MD13G1160800 |  | 1.562 | 6.74707E-18 |
| MD13G1160600 |  | 2.289 | 2.05576E-20 |
| MD16G1161100 |  | 1.004 | 5.69697E-07 |
| MD13G1161000 |  | 4.059 | 0.000492837 |
| MD16G1161400 |  | 3.307 | 1.52149E-05 |
| MD16G1160400 |  | 4.19 | 1.6426E-10 |
| MD16G1160300 |  | 2.205 | 5.14623E-11 |
| MD15G1275500 | SABP2;  salicylic acid-binding protein 2-like | 2.509 | 3.17426E-15 |
| MD06G1182800 | EDS1; enhanced disease susceptibility 1 | 1.951 | 3.78344E-11 |
| MD14G1188600 |  | 1.4 | 2.03925E-28 |
| MD06G1182600 |  | 2.254 | 9.32583E-28 |
| MD15G1136300 | PAD4; lipase-like PAD4 isoform X1 | 1.713 | 1.65709E-12 |
| MD10G1266400 | WRKY71; probable WRKY transcription factor 71; Involved in the activation of salicylic acid biosynthesis genes ICS1 and PBS3. | 2.632 | 6.47245E-05 |
| MD05G1290300 |  | 2.393 | 1.45807E-10 |
| MD17G1138100 |  | 2.438 | 2.36207E-07 |
| MD09G1150700 |  | 2.609 | 4.31297E-08 |
| MD03G1128900 | SOD2;  superoxide dismutase [Cu-Zn] | -1.93 | 1.78468E-25 |
| MD11G1148200 |  | -1.483 | 1.87097E-18 |
| MD07G1248400 | ERF2;  Ethylene-responsive transcription factor 2-like | 1.292 | 0.021684696 |
| MD06G1120000 | MYC2-1; transcription factor MYC2 | (RefSeq) transcription factor bHLH14 (A) | 2.298 | 1.20967E-16 |
| MD14G1137200 |  | 4.619 | 0.006892915 |
| MD10G1328100 | ACO1;  Full=1-aminocyclopropane-1-carboxylate oxidase 1; Short=ACC oxidase 1 | 2.459 | 0.025655066 |
| MD10G1281600 | SAG12; Senescence-specific cysteine protease SAG12 | 2.233 | 0.007624296 |
| MD05G1288200 |  | 2.494 | 0.000207353 |
| MD10G1281700 |  | 2.671 | 1.15315E-06 |
| MD05G1287900 |  | 3.293 | 0.015292258 |
| MD12G1216000 | ATG18a-like;  autophagy-related protein 18a-like | 2.613 | 0.001757706 |

**Table S3 Levels of phytohormones and metabolites related to SA and antioxidant activity in aerial parts from Ri-1 and GL-3.**

Phytohormones were shown as absolute levels (ng g^-1^ fresh weight). Metabolites are showed as relative levels. Data were normalized to the mean values of GL-3. Values are means ± SD (n = 3, three biological replicates). ** indicates P < 0.01; * < 0.05.

| Compounds | GL-3 | Ri-1 | Class |
| --- | --- | --- | --- |
| SA | 13.817 ± 7.5964 | 98.5933 ±12.921** | SA |
| JA | 2.325 ± 0.7246 | 1.7707 ± 0.1798 | JA |
| H2JA | 2.8017 ± 0.0335 | 2.919 ± 0.0916 |
| JA-ILE | 5.9787 ± 1.4542 | 5.889 ± 0.5133 |
| IAA | 1.6463 ± 0.1008 | 1.0797 ± 0.0465 ** | Auxin |
| ME-IAA | 1.5257 ± 0.0491 | 1.5477 ± 0.0354 |
| ICA | 1.0637 ± 0.1451 | 1.1293 ± 0.0621 |
| Benzoic acid | 1.00 ± 0.43 | 2.71± 0.45** | SA derivatives |
| 4-Hydroxybenzoic acid | 1.00 ± 0.76 | 6.62 ± 1.38** |
| Phenyl salicylate | 1.00 ± 0.27 | 2.02 ± 0.51* |
| Glutathione oxidized | 1.00 ± 0.30 | 1.52 ± 0.95 | Amino  acid  derivatives |
| Glutathione reduced form | 1.00 ± 0.16 | 1.11 ± 0.20 |
| S-(methyl)glutathione | 1.00 ± 0.13 | 1.40 ± 0.15 |
| *p*-Coumaric acid | 1.00 ± 0.15 | 1.04 ± 0.18 | Hydroxycinnamoyl derivatives |
| *Myo*-inositol | 1.00 ± 0.0316 | 0.614 ± 0.112** | Sugars and  sugar  alcohols |
| L-ascorbate | 1.00 ± 0.13 | 1.04 ± 0.22 | Vitamins |

**Table S4 Expression level of ET biosynthetic genes in GL-3 and MdUGT88F1-RNAi apple lines by RNA-seq analysis.**

Data are shown as means of FRKM (fragments per kilobase of transcript per million mapped reads) ± SD (n = 3, three biological replicates).

|  | Gene ID | GL-3 | Ri-3 | Ri-6 |
| --- | --- | --- | --- | --- |
| MdACO1 | MD10G1328100 | 0.0445 ± 0.078 | 0.084 ± 0.028 | 0.043 ± 0.075 |
|  | MD05G1354000 | 47.075 ± 15.859 | 31.324 ± 11.764 | 19.251 ± 11.149 |
| MdACS1 | MD15G1302200 | 0.014 ± 0.0241 | 0.012 ± 0.020 | 0 |
| MdACS3a | MD02G1073900 | 0 | 0.016 ± 0.027 | 0 |
| MdACS4 | MD00G1167500 | 0 | 0.016 ± 0.027 | 0 |
|  | MD01G1070400 | 0 | 0 | 0.015 ±0.026 |
| MdACS5 | MD14G1111500 | 0 | 0.079 ± 0.084 | 0.088 ± 0.093 |
|  | MD06G1090600 | 0.419 ± 0.399 | 1.235 ± 0.584 | 0.667 ± 0.693 |
| MdACS6 | MD01G1186400 | 4.732 ± 0.136 | 3.987 ± 0.247 | 3.682 ± 0.202 |
|  | MD04G1076200 | 48.456 ± 11.535 | 38.895 ± 1.731 | 32.287 ± 4.004 |

**Table S5 Primers used in this study**

| Primer Name | Primer Sequence (5’ to 3’) | Purpose |
| --- | --- | --- |
| MdMIPS1-F | ATGTTCATCGATAGCTTCAAGGTAGAGT | Clone *MdMIPS1* full length |
| MdMIPS1-R | TCACTTGTATTCCAAGATCATGTTGTTC |
| MdMIPS1-RNAi-F | GGGGACAAGTTTGTACAAAAAAGCAGGCTTTCAAGGTAGAGTCCCCAAACGT | Plasmid construction of *MdMIPS1/2* RNAi |
| MdMIPS1-RNAi-R | GGGGACCACTTTGTACAAGAAAGCTGGGTTATGTCATCTGGGTTCACCATAGG |
| L1 | CCAACTTTGTACAAAAAAGCAGGCT | Genomic-PCR identification of *MdMIPS1/2* silencing apple lines |
| L2 | CCAACTTTGTACAAGAAAGCTGGGT |
| 2300-MdMIPS1-F | GGATCCGCCACCATGTTCATCGATAGCTTCAA | Plasmid construction of pCambia2300-*MdMIPS1* |
| 2300-MdMIPS1-R | GGTACCTCACTTGTATTCCAAGATCATGTTGTTC |
| 2300F | GAGAACACGGGGGACTCTAGA | Genomic-PCR identification of pCambia2300-meidated transgenic apples |
| 2300R | CGATCGGGGAAATTCGAGCTC |
| qEF-F | ATTCAAGTATGCCTGGGTGC | RT-qPCR analysis |
| qEF-R | CAGTCAGCCTGTGATGTTCC |
| qMdMIPS1-F | AGGCCAAGTTCCACTCATTCCAC | RT-qPCR analysis |
| qMdMIPS1-R | CAAGCCCTCAGTATGTTCTCCAG |
| qMdMIPS2-F | GATTGTGGGGCTAAACGACACCG | RT-qPCR analysis |
| qMdMIPS2-R | ACTGTTTCTCCTAATTGCCAAATCAATGAGT |
| qPR1-F | CACAAAACTACGCCAACCAA | RT-qPCR analysis |
| qPR1-R | AGCACACGAGTTCGACTCATAA |
| qPR2-F | TGGGACTCGATACCCTAGCCTCT | RT-qPCR analysis |
| qPR2-R | GCTTGATCACCACCTTCAGAAGGC |
| qPR4-F | CCACCTCTACAATCCACAGCAAAAC | RT-qPCR analysis |
| qPR4-R | GTCCGCAAAAGGCAGTCCATCCA |
| qPR5-F | AACTAGCATCCAAAGCTAGCC | RT-qPCR analysis |
| qPR5-R | CCACAGTCTGCAGTTTCACAAG |
| qPR8-F | GGAGGGGCCACCGAAACTTACTCT | RT-qPCR analysis |
| qPR8-R | TCGAAATCGACGCCGTCCAAAACC |
| qACS4-F | GACACACTTGAAAGCCTTG | RT-qPCR analysis |
| qACS4-R | TTTTCGGCCAATGTTGACC |
| qACS5-F | GGGGTTCCCTGGCTTCAGAG | RT-qPCR analysis |
| qACS5-R | GTGGCTCGAACAAGAGGCG |
| qACO1-F | GTTCTACAACCCAGGCAACG | RT-qPCR analysis |
| qACO1-R | TCTCAGAGCTCAGGCAGTTG |
| qSAG12-F | CGATGCTAGCGGTTCCGATTTCC | RT-qPCR analysis |
| qSAG12-R | CCCCATTCTGCACCCCATGAGTT |

**
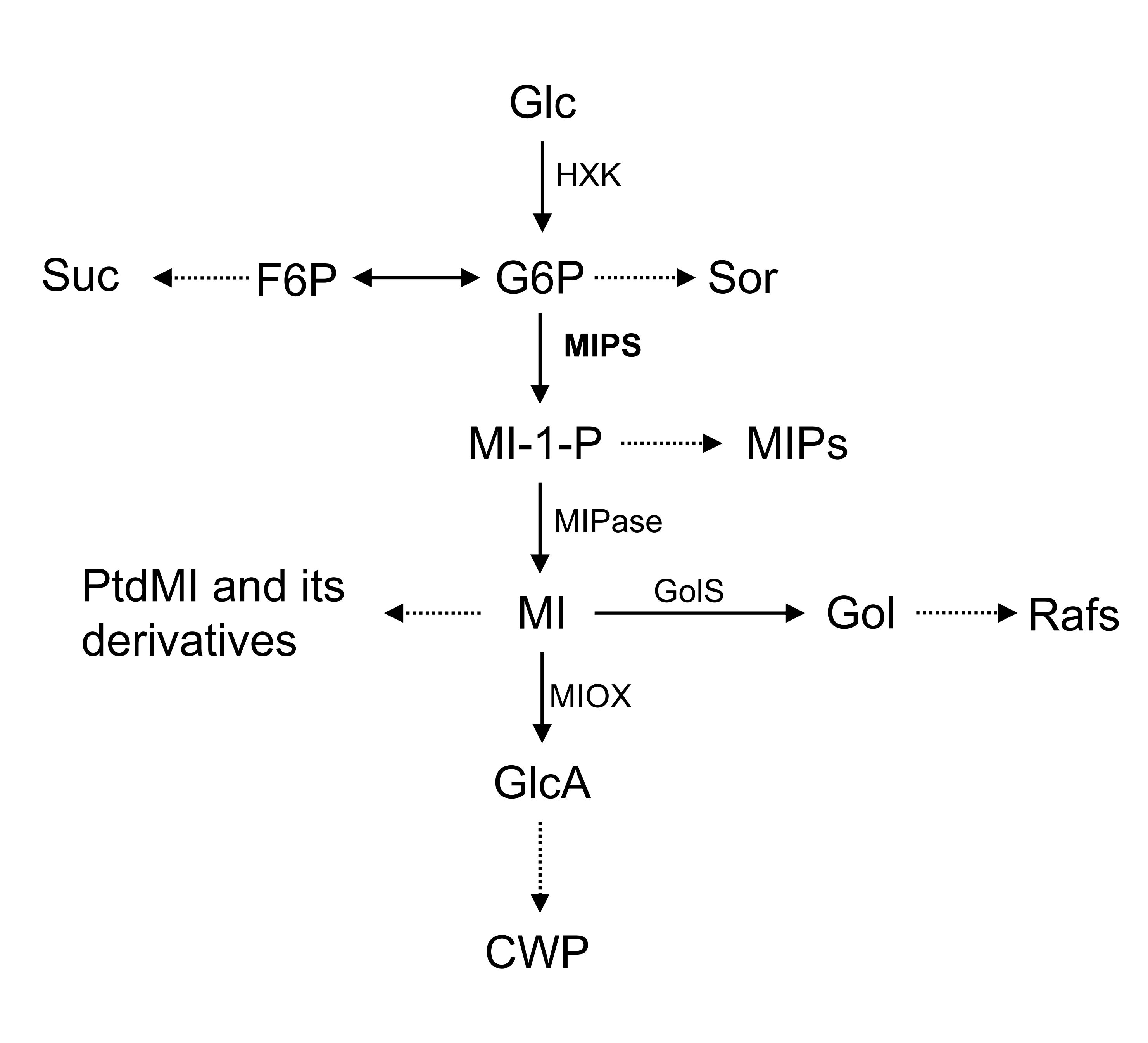
**

**Fig. S1 Biosynthetic pathways of *myo*-inositol, its derivatives, and selected sugar alcohols.** HXK, hexokinase; MIPS, *myo*-inositol-1-phosphate synthase; MIPase, *myo*-inositol monophosphatase; MIOX, *myo*-inositol oxygenase; Glc, glucose; G6P, glucose-6-phosphate; F6P, fructose-6-phosphate; Suc, sucrose; Sor, sorbitol; MI, *myo*-inositol; MI-1-P, *myo*-inositol-1-phosphate; PtdMI, phosphatidylinositol; Gol, galactinol; Rafs; raffinose-family oligosaccharides; GlcA, D-glucuronic acid; CWP, cell wall polysaccharides; and GolS, galactinol synthase. Solid and dashed arrows indicate direct reactions and multiple-step-reactions, respectively.


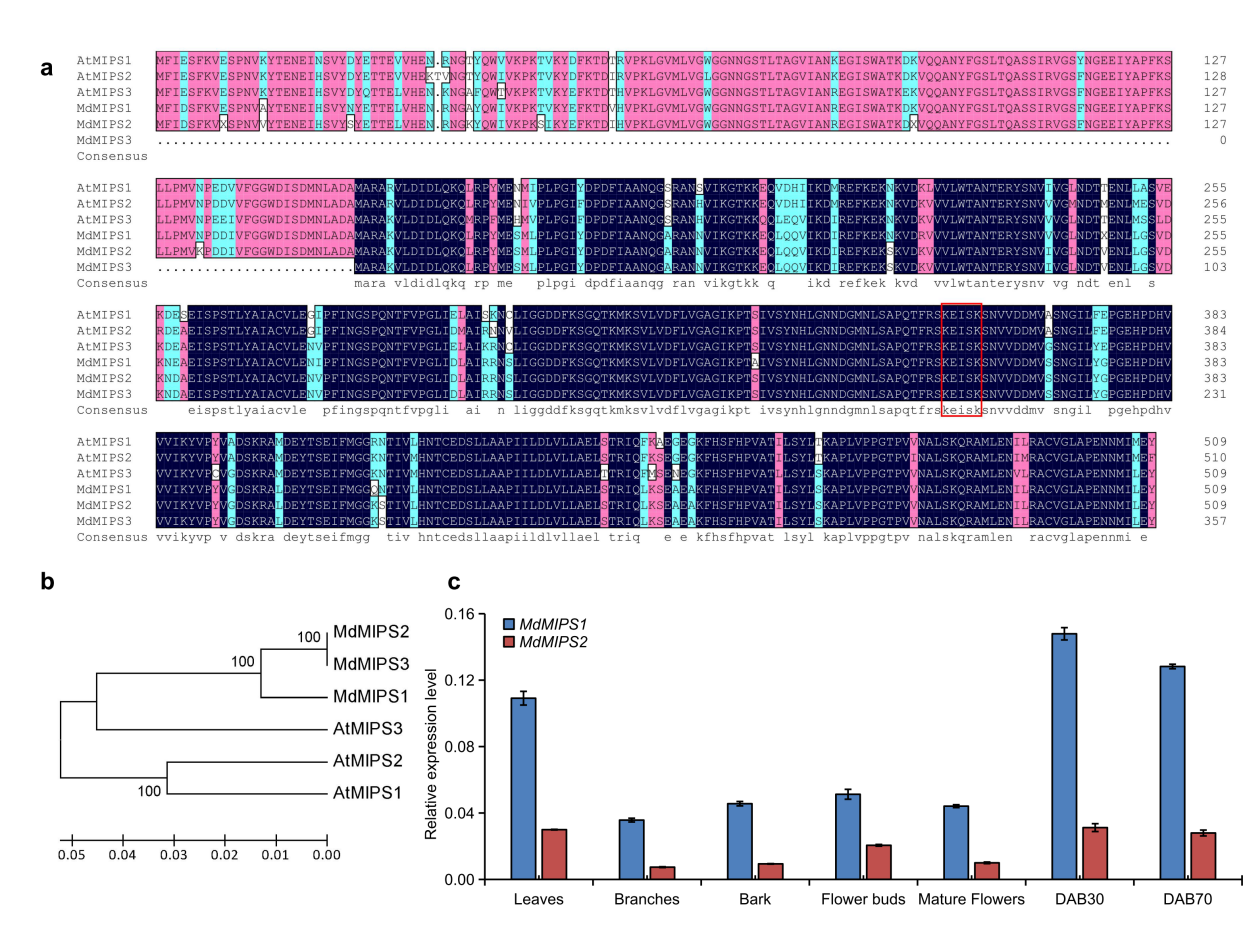


**Fig. S2 Identification of *MIPS* genes in the apple genome.**

**(a)** Alignment of protein sequences between AtMIPSs and MdMIPSs; Black, red, and blue shading represents identical sequences, >75%, and >50% sequence similarity. **(b)** Phylogenetic analysis of MIPSs from apple. **(c)** Expression patterns of *MdMIPS1* and *MdMIPS2* in different tissues from ‘Royal Gala’. Data are represented as means ± SD (n = 3, three biological replicates). The red box indicates a conserved pentapeptide. DAB30 and DAB70 indicate 30 and 70 days after bloom, respectively.





**Fig. S3 Identification and characterization of transgenic apple lines.**

**(a)** Genomic-PCR identification; Marker, DL2000; VC, vector control. **(b)** Transcript levels of *MdMIPS1* and *MdMIPS2* in the leaves of GL-3 and transgenic candidates from tissue culture. **(c)** Phenotypes of transgenic apple lines under MI-depleted MS conditions. Data are means ± SD (n = 3, three biological replicates). *** indicates significant expression differences from GL-3 at P < 0.001.


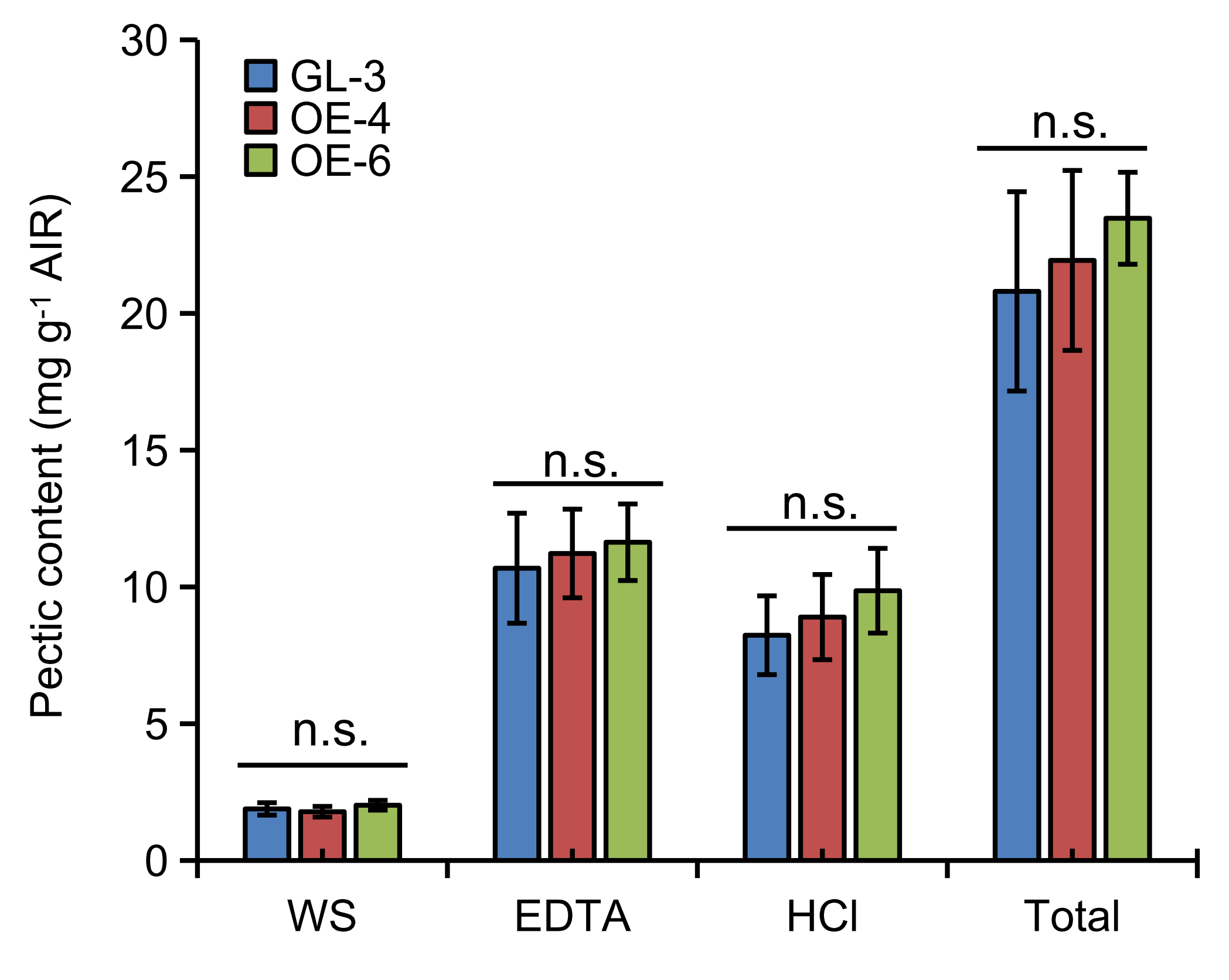


**Fig. S4 Pectin composition in GL-3 and *MdMIPS1*-overexpressing apple stems under greenhouse conditions.**

Data are means ± SD (n = 7, seven biological replicates). n.s. indicates no significant differences compared with GL-3. WS, EDTA, and HCl represent crude cold water, EDTA, and HCl soluble fractions, respectively. Four-month-old plants were used.


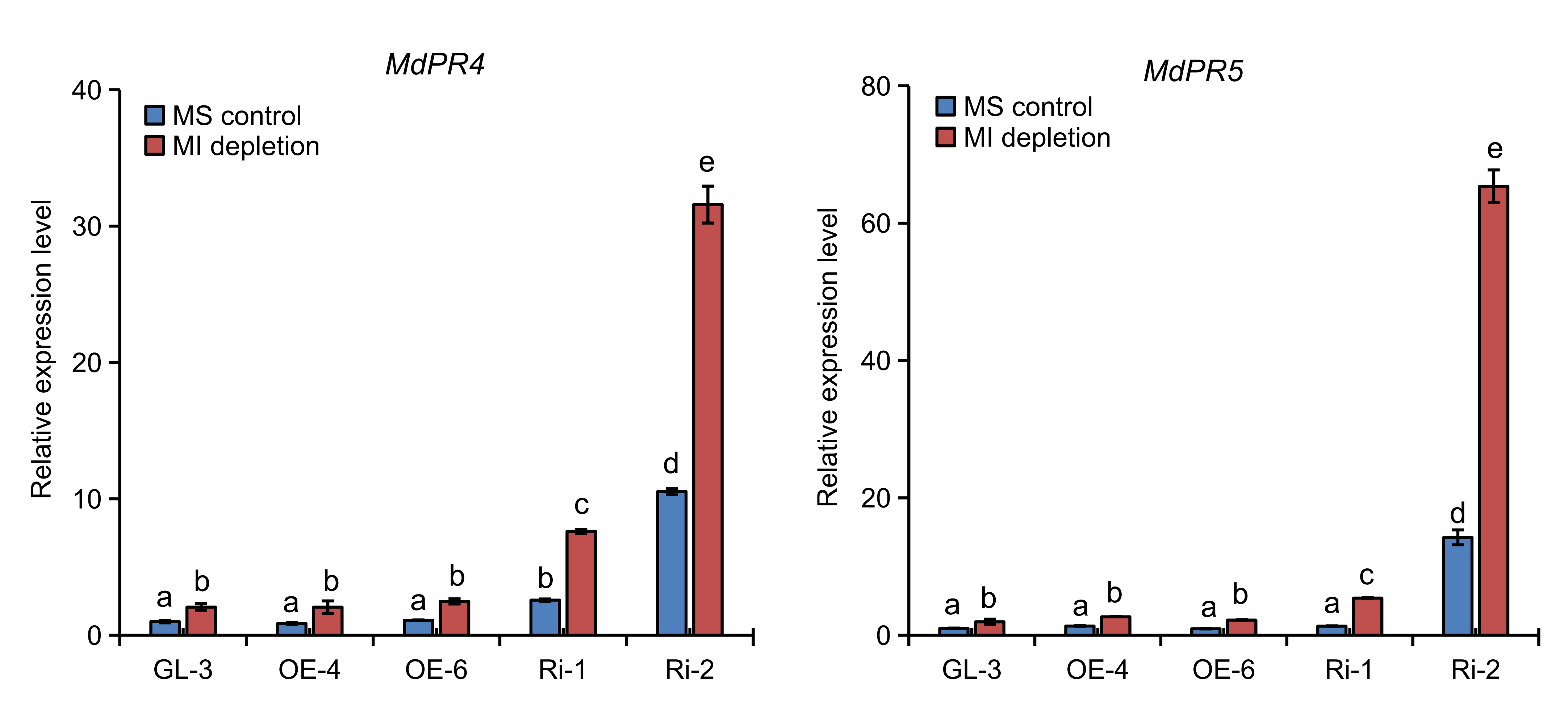


**Fig. S5 Changes in *MdPR4* and *MdPR5* expression in transgenic apple lines in response to MI-depletion.**

Data are means ± SD (n = 3, three biological replicates). Values not represented by the same letter are significantly different (P < 0.05).





**Fig. S6 Effects of ET signaling on ROS accumulation and SA signaling in MdMIPS1-RNAi apple lines.**

Effects of 1-MCP application on H_2_O_2_ accumulation **(a)**, anti-O_2_^-^ **(b)**, SOD **(c)** activity, and *MdSAG12* **(d)**, *MdPR1* **(e)** and *MdPR5* **(f)** expression. Data are means ± SD (n=3, three biological replicates). Values not represented by the same letter are significantly different (P < 0.05).





**Fig. S7** **Effects of 1-MCP application on GL-3 and Ri-1 growth.**

Data are means ± SD. Values not represented by the same letter are significantly different (P < 0.05).


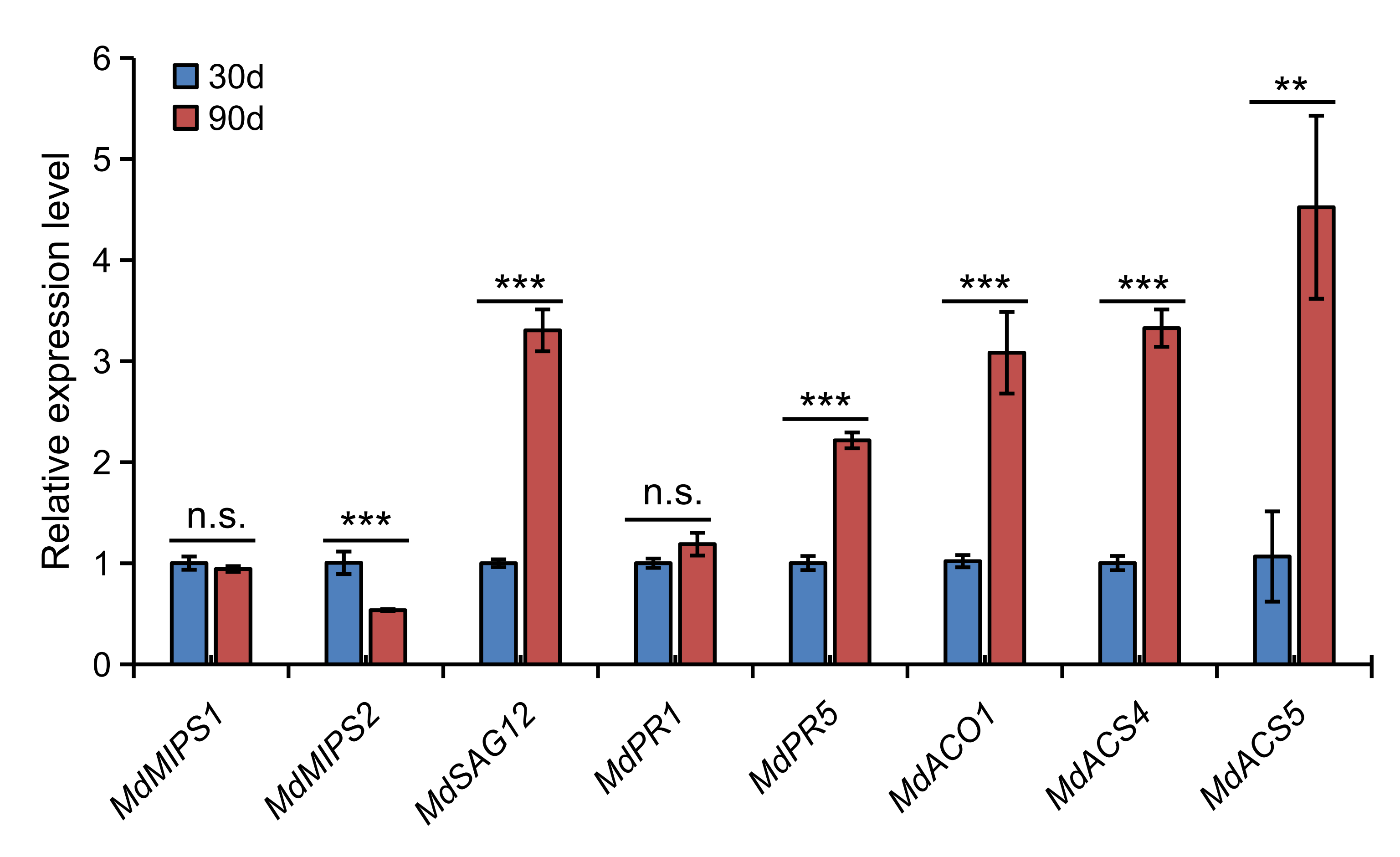


**Fig. S8 Expression levels of *MdMIPSs*, *MdSAG12*, ET biosynthetic genes, and *MdPRs* in GL-3 leaves of different ages.**

Data are means ± SD (n = 3, three biological replicates). Values not represented by the same letter are significantly different (P < 0.05). *** and ** indicates significant expression differences at P < 0.001 and 0.01, respectively. n.s. indicates no significant differences.

**

**

**Fig. S9 *Valsa* canker resistance in GL-3 and transgenic apple lines**.

Evaluation results of *Valsa* canker resistance in the transgenic apple lines and GL-3 by leaf (15-d-old, **a** and **b**) and stem (5-month-old, **c** and **d**) inoculation. Data are means ± SD; n indicates biological replicates, one leaf or stem from each plant was used. n.s. indicates no significant differences compared with GL-3. Bar = 1 cm for **a**.
